# Shock-recovered maskelynite indicates low-pressure ejection of shergottites from Mars

**DOI:** 10.1126/sciadv.adf2906

**Published:** 2023-05-03

**Authors:** Jinping Hu, Paul D. Asimow, Yang Liu, Chi Ma

**Affiliations:** ^1^ Division of Geological and Planetary Sciences, California Institute of Technology, Pasadena, CA 91125, USA.; ^2^ Jet Propulsion Laboratory, California Institute of Technology, Pasadena, CA 91109, USA.

## Abstract

Diaplectic feldspathic glass, commonly known as maskelynite, is a widely used impact indicator, notably for shergottites, whose shock conditions are keys to their geochemistry and launch mechanism. However, classic reverberating shock recovery experiments show maskelynitization at higher shock pressures (>30 gigapascals) than the stability field of the high-pressure minerals found in many shergottites (15 to 25 gigapascals). Most likely, differences between experimental loading paths and those appropriate for martian impacts have created this ambiguity in shergottite shock histories. Shock reverberation yields lower temperature and deviatoric stress than single-shock planetary impacts at equivalent pressure. We report the Hugoniot equation of state of a martian analog basalt and single-shock recovery experiments, indicating partial-to-complete maskelynitization at 17 to 22 gigapascals, consistent with the high-pressure minerals in maskelynitized shergottites. This pressure explains the presence of intact magmatic accessory minerals, used for geochronology in shergottites, and offers a new pressure-time profile for modeling shergottite launch, likely requiring greater origin depth.

## Teaser

Experiments on shock amorphization of plagioclase in Mars-like basalt reconcile the pressure scale for martian meteorites.

## INTRODUCTION

The feldspar-to-maskelynite transformation is one of the most widely observed shock-metamorphic features in affected rocks (*1*). Although the original 19th century identification of maskelynite in the Shergotty meteorite as a new mineral was inaccurate because of the shortcomings of 19th century analytical techniques (*2*, *3*), the term has subsequently come to describe isotropic feldspathic glass created by pressure-induced solid-state transformation (diaplectic glass) (*4*), whereas quenched feldspathic melt is empirically referred to as normal glass. The shock pressure (*P*)–temperature (*T*)–pulse duration time (*t*) conditions indicated by the formation and preservation of maskelynite offer essential constraints on the thermal (*5*) and launch history (*6*–*8*) of shergottites, a subgroup of martian meteorites in which plagioclase is always partially or fully maskelynitized, supposedly due to the shock experienced during impact-driven acceleration to the escape velocity of Mars (*9*, *10*).

**Fig. 1.**
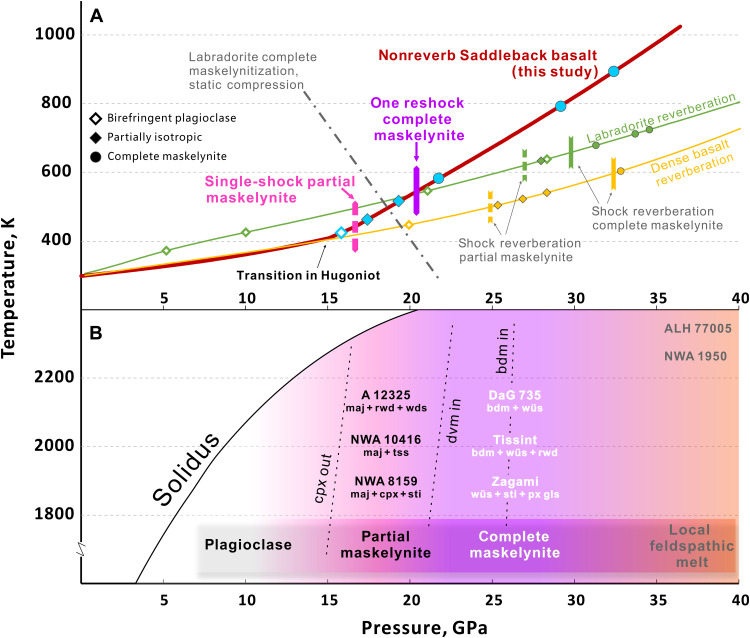
Pressure-temperature of maskelynite formation in experiments and martian meteorites. (**A**) Solid curves are pressure-temperature (*P*-*T*) estimates of shock experiments. The curve of Saddleback basalt (An_65_ phenocrysts) is from this study (dark red curve); the sharp change in slope corresponds to the phase change to a denser state. The interpolated thresholds for partial and complete maskelynitization are indicated by the pink dashed and solid purple vertical markers on the curve. The curves for single An_65_ labradorite crystal (green) and dense basalt with An_68_ labradorite (yellow) show *P*-*T* conditions calculated from the data of reverberating recovery experiments (*11*–*13*, *37*) and corresponding maskelynitization thresholds (vertical dash and solid markers). Open diamond, solid diamond, and solid circle on each curve indicate birefringent plagioclase and partial and full maskelynite, respectively, for the set of experiments. The tilted dot-dash line is the labradorite-maskelynite phase boundary from static compression (*21*). (**B**) Previously inferred stability fields of mineral assemblages in various shergottites, including ringwoodite (rwd), wadsleyite (wds), majorite (maj), stishovite (sti), tissintite (tss), clinopyroxene (cpx), davemaoite (dvm), bridgmanite (bdm), wüstite (wüs), pyroxene glass (px gls) and ferropericlase (fp) (*16*, *17*, *52*–*54*). The vertical positions are schematic, because shocked meteorites experience complex, heterogeneous, evolving temperature conditions. Allan Hills (ALH) 77005 and Northwest Africa (NWA) 1950 do not contain HP minerals but commonly have quenched feldspar glass (*18*). The pink-purple-orange background colors indicate plagioclase-maskelynite-melt transition from this study.

The shock conditions required for maskelynitization have been investigated in shock recovery experiments (*4*, *11*–*15*). These studies converge on a range from 26 to 32 GPa as the threshold for partial to complete amorphization of calcic plagioclase (Fig. 1) (*9*). However, shock pressures exceeding 30 GPa for maskelynitized shergottites are inconsistent with the increasing recognition that their high-pressure (HP) mineral assemblages have stability fields limited to <25 GPa (*16*, *17*). Moreover, shock pressure substantially above 30 GPa is expected to enhance local melting (*18*) and cause transformation and reversion of baddeleyite to and from HP polymorphs with the potential for Pb loss (*19*, *20*), which may be inconsistent with the magmatic crystallization U-Pb ages commonly recorded by untransformed baddeleyite grains found directly adjacent to maskelynite in shergottites (*5*). This discrepancy remains an impediment to understanding the shock disturbances and launch process experienced by shergottites. More nuanced compression studies show that peak pressure, pulse duration, temperature, strain rate, and deviatoric stress are all important factors affecting maskelynitization (*21*–*24*). Full amorphization of calcic plagioclase requires 32 GPa in 20-ns laser shocks (*24*) but <10 GPa in longer-pulse, shockless rapid compression (*23*). Hence, if the presence of maskelynite is to offer an accurate peak pressure for shergottites, then the thermobarometer needs to be based on experiments that resemble, as closely as possible, the *P*-*T*-*t*–$\dot{\epsilon }$ (strain rate) path associated with natural impacts into shergottite-like targets on Mars.

In natural impacts, initial loading to HP commonly occurs in a single step from ambient to Hugoniot conditions (*25*). Two-wave loading due to phase transitions or shock reflections from impedance contrasts (like metal grains) is possible but should be localized (*26*). Multistep loading to peak pressure is rare in nature and was not the path leading to pervasive maskelynite in shergottites. However, classic shock recovery experiments commonly use multiple shock reflections across a low-impedance sample embedded in a high-impedance chamber, whereby the sample “rings up” to a peak pressure equal to the shock pressure in the chamber material (fig. S1). The advantage of this reverberation technique is that peak pressure in the sample is independent of the sample’s Hugoniot equation of state (EoS), which may be unknown and complex to model. However, loading to a given peak pressure by reverberation also results in lower energy, temperature, and deviatoric stress than single-shock loading, thereby differing from the *P*-*T*-*t*-$\dot{\epsilon }$ path of natural shocks and making it harder to produce pronounced shock metamorphism (*27*). This may be the primary cause of the pressure gap between the threshold for maskelynite formation in experimentally shock recovered samples and in naturally affected shergottites (*9*, *17*). Moreover, many previous experimental studies used plagioclase single crystals (*12*, *13*) as starting materials. Single crystals have zero porosity and follow a lower-temperature path than the likely target materials on Mars, where shock melt pockets record at least local high temperatures (*28*). In this study, we develop a shock recovery setup to generate well-defined single-shock loading paths, resembling the *P*-*T*-*t*-$\dot{\epsilon }$ path of natural impacts on Mars. On the basis of results from a Mars rock simulant (a slightly porous natural basalt), we propose an improved calibration of the maskelynitization conditions in shergottites.

## RESULTS AND DISCUSSION

### Hugoniot EoS of basalt

**Fig. 2.**
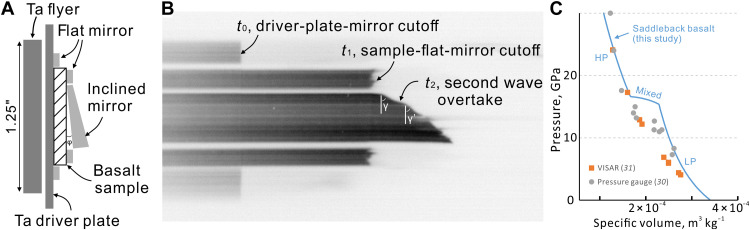
Setup and results of Hugoniot EoS measurement of Saddleback basalt. (**A**) Ta flyer and target assembly consisting of Ta driver plate, basalt sample disc, and mirrors silvered on the front (left) side to reflect light to streak camera. Flyer-driver-sample sizes are to scale. (**B**) Color-inverted streak camera image, with time increasing to the right and vertical axis corresponding to positions across the sample as shown in (A). Total streak duration is 4 μs. The end of each dark streak shows a shock wave hitting the mirror at that position and time. *t*_0_ and *t*_1_ correspond to the time of shock wave entering the sample and reaching the back of the sample. Inclined mirror cutoff indicates the timing of decompressed material hitting the mirror at free surface velocity, determined by slope angle γ. The change in slope at time *t*_2_ is caused by a second shock wave overtaking the first arriving wave. Irregular cutoffs result from the heterogeneity of porosity and mineralogy of the basalt at millimeter scale. (**C**) The fitted Hugoniot in pressure-volume (*P*-*V*) space. The piecewise curve indicates low pressure (LP), mixed phase, and high pressure (HP) regimes. Curvature of the LP regime does not affect the pressure calculations for our recovery shots. The Hugoniot data of Kinosaki basalt measured by piezoresistive gauges (*30*) and velocity interferometer (VISAR) (*31*) show similar complication, although not exactly matching Saddleback basalt (fig. S2 and S3).

To approach shock conditions matching shergottites, we used Saddleback basalt, the source of Mojave Mars Simulant (*29*), which is rich in phenocrysts of An_65_ labradorite (table S1). To design recovery experiments that achieve single-shock loading and to know the pressure precisely in such experiments, we measured the Hugoniot EoS of Saddleback basalt (Fig. 2 and Materials and Methods), i.e., the family of shock states achieved by shocks of varying strength into this starting material. The shock velocity (*U*_s_) and particle velocity (*u*_p_) of Saddleback basalt were measured using the inclined mirror technique (see Materials and Methods); the data are shown in pressure-volume (*P*-*V*) space in Fig. 2.

In the absence of phase change or elastic-plastic transition during shock, the *U*_s_-*u*_p_ Hugoniot is empirically linear and the *P*-*V* Hugoniot is derivable. In contrast, Saddleback basalt shows a fast low-pressure (LP) wave and a slow HP wave (Fig. 2C), interpreted to be a density transition to a denser state, which results in a piecewise Hugoniot with LP, HP, and mixed regimes (Fig. 2C). The density transition occurs in the range of 15.4 to 16.6 GPa. Although not all published Hugoniots of basalts show this transition clearly, a phase change is observed in Kinosaki basalt at 13 to 18 GPa (*30*). Even in a study that found final shock states along a nearly linear Hugoniot (Fig. 2C) (*31*), time-resolved wave velocities indicate stepped pressure rise and complex wave structures in this range (fig. S3). Our experimental determination of the Hugoniot EoS of Saddleback basalt enables precise interpretation of shock pressures in our recovery experiments.

### Shock-recovered maskelynite in basalt

**Fig. 3.**
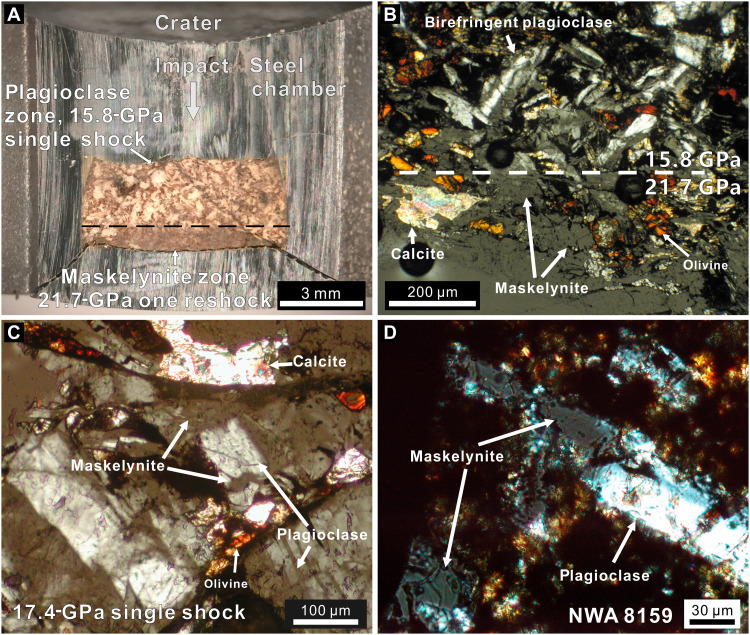
Photos of shock-recovered Saddleback basalt. (**A**) Thick section of S1240 sample in steel chamber with a region that experienced a single shock to 15.8 GPa and a region that experienced one reflected shock to 21.7 GPa, with boundary indicated by a dashed line. Plagioclase in the single-shocked region shows its original white color, but the maskelynite in the reshocked region is transparent and displays deformed shapes. Two oblique fractures propagated from the deformed thread of the rear chamber cap. (**B**) Nonorthogonal (87° to 88°) cross-polarized light (xpl) micrograph of the pressure transition region of S1240 in thin section. All plagioclase grains transformed to maskelynite (isotropic) in the reshocked zone. (**C**) Nonorthogonal xpl image of partial maskelynite in region of recovered sample S1244 that experienced single shock to 17.4 GPa. Plagioclase grains are 30 to 40% maskelynitized. The nonisotropic portions show lower birefringence than single-shocked grains in S1240. (**D**) Nonorthogonal xpl image of partial maskelynite in shergottite NWA 8159 (*17*).

Seven recovery experiments span the transition between density regimes along the Hugoniot (table S3). For three single/double-shock experiments, we used sample/flyer thickness ratios greater than 2 to prevent reverberation (see Materials and Methods); this allows time for at most one shock reflection to partially transit the sample before a release wave arrives to attenuate the shock. The sample region that released after only one shock transit and the region that released after one shock reflection can be identified unambiguously in this geometry (Fig. 3A).

S1240 is the shot with the lowest impact velocity. The front of the sample experienced a single loading pulse to 15.8 GPa and maintained for 1.8 μs before release wave arrival (fig. S5). The single-shocked central front area of the recovered sample contains almost all birefringent plagioclase (Fig. 3B) and shows a white color in thick section (Fig. 3A). The back of the sample in the same experiment experienced one reshock from the steel back wall and reached 21.7 GPa. This reshocked area displays isotropic maskelynite (Fig. 3B) and transparent grains in thick section (Fig. 3A). The visual boundary between zones of amorphized and crystalline plagioclase is plainly visible in both thin and thick sections (dashed line in Fig. 3, A and B) and coincides with the intersection of the reshock and release wave.

Two more recovery experiments help to refine the nature of the glass transition upon single- and double-shock loading. S1244 captures the onset of partial maskelynitization in the front single-shock region at 17.4 GPa (Fig. 3C). Multiple plagioclase grains in this region are divided into areas that are isotropic and areas that display curved twin planes and low birefringence (Fig. 3C). All plagioclase grains in the reshocked region (peak pressure: 29.2 GPa) are amorphized. The corners of the capsule, which experience edge effects and strong shear heating, contain complete maskelynite that formed at poorly known *P*-*T* conditions somewhat different from the central part (fig. S6). S1245, with slightly higher impact velocity and peak pressure of 19.3 GPa in the single-shock region, shows a noticeably higher degree of partial maskelynitization than S1244. Some large (~500-μm) feldspathic domains are completely isotropic, and other plagioclase grains show very low birefringence that makes twinning unobservable. Most feldspar domains look transparent in thick section (fig. S7), resembling the fully maskelynitized regions in other recovered samples (e.g., Fig. 3A). To ensure the correct identification of weakly birefringent plagioclase, we used electron backscatter diffraction (EBSD) to map out feldspathic domains with diffraction patterns (fig. S7). Diffraction band contrast maps of S1245 indicate that some feldspar domains in the back of the single-shock region, whose shock pulse is 0.5 μs shorter than that of the sample front (fig. S5), still retain some level of crystalline structure, despite showing very low birefringence in cross-polarized light. In both S1244 and S1245, the single-shock regions contain scarce instances of glass displaying flow and schlieren features, presumed to be quenched from local melting of plagioclase (plus some pyroxene). This feldspathic normal glass shows notably lower fracture density than the surrounding maskelynite or plagioclase (figs. S6 and S7), indicating viscous relaxation of shear stress before shock release.

The results of the three thick sample recovery shots spanning the glass transition interval along the Hugoniot demonstrate the onset of maskelynitization occurs around 17 GPa, with maskelynite becoming predominant above 19 GPa and complete transition by 22 GPa. The pressures are slightly higher than the transition point in the Hugoniot (Fig. 1), likely because some excess pressure is needed to preserve the amorphization upon recovery (*24*, *32*). The onset pressure of maskelynitization for single shocks of our target material is 17 GPa, much lower than the 25 to 27 GPa in reverberation experiments (Fig. 1). Our thin-sample experiments replicated previous results, showing that reverberation causes complete maskelynitization of Saddleback basalt at around 30 GPa or above (table S3 and fig. S8).

### Low shock pressure and temperature of shergottites

The pressure threshold for conversion of plagioclase to maskelynite is not a simple function of peak pressure but depends on the *P*-*T*-*t*-$\dot{\epsilon }$ loading path (*21*, *23*, *24*). Evaluation of the peak pressure of shergottites therefore requires experiments that approach the conditions of natural impacts on Mars. Unfortunately, natural impacts, laboratory shocks (propellant- or laser-driven), static, and rapid compression experiments all populate different regimes in *P*-*T*-*t*-$\dot{\epsilon }$ space. Planetary impacts related to shergottites are thought to involve pulses of 10^−3^ to 10^−2^ s duration and strain rates greater than ~10^5^ s^−1^ (*17*, *33*–*35*). Reproducing that duration and strain rate simultaneously is challenging. Shock recovery experiments provide the correct strain rate but a shorter pulse duration, <10^−5^ s (fig. S5), whereas anvil compression provides longer pulse durations but much lower strain rates, <10^−1^ s^−1^ (*21*, *23*). Our propellant shock experiments with microsecond pulses demonstrate maskelynitization pressures intermediate between estimates from laser shocks of 20-ns duration (*24*) and anvil compression experiments lasting at least seconds (*23*), suggesting a negative correlation between transformation pressure and pulse duration. Therefore, the pressure thresholds (17.4 to 21.7 GPa) observed in our experiments are probably slightly higher than the actual pressure of partially maskelynitized shergottites (Fig. 3D) launched by martian impacts. In other words, our experiments set a new upper bound for the maskelynitization thresholds of calcic plagioclase in natural impacts. This threshold also applies to most terrestrial impact sites, whose pulses last >10^−3^ s.

Increasing temperature favors maskelynite formation at lower pressure. Static compression experiments observe this effect (Fig. 1) (*21*). Likewise, preheating of basalt to 1073 K lowers the threshold for partial maskelynitization in reverberation experiments from >26 to ~22 GPa (*36*). The higher shock temperatures achieved by single shocks compared to previous reverberation shock paths might therefore explain the observation of maskelynite formation at ~17 GPa. However, calculated shock temperatures for single-shock and reverberating shock loading paths (Fig. 1A) are negligibly different in the range where we find the onset of maskelynite formation, becoming more different only above 30 GPa. Although one-step loading of a target with the appropriate porosity does offer a better match to the shock temperatures experienced by shergottites than reverberation shocking single crystals, temperature does not appear to solely explain the markedly lower maskelynitization threshold in our experiments compared to reverberation studies.

Another key difference between single and reverberating shock loading is the magnitude of deviatoric stress experienced by the sample. Reverberating shocks that peak at 25 to 30 GPa typically have first shock fronts with pressure amplitudes of ~10 GPa that overdrive the Hugoniot elastic limit (HEL; ~5 GPa) by about 5 GPa before material failure (*37*). It is likely that subsequent shocks raise the pressure in a nearly hydrostatic fashion without material strength effects and cause limited increases in deviatoric stress. In contrast, a single shock directly to the peak pressure creates transient deviatoric stress several times larger by overdriving the HEL more strongly (*38*). Large deviatoric stresses are likely to facilitate LP maskelynitization (*39*). Hydrocode simulation also shows that shear stress varies temporally and spatially for regions of the same peak pressure during impact cratering, which plays an important role in producing meter-scale features such as shutter cones (*40*). Hence, the single-shock experiments better resemble this aspect of the *P*-*T*-*t*-$\dot{\epsilon }$ path of martian impacts. In an actual planetary impact, the longer duration (*33*), shock turbulence (*41*), and extensive shear flows (*42*) may all contribute to further lowering the pressure threshold for maskelynitization.

Raman spectra of feldspars in shergottites demonstrate broadening of diagnostic peaks with increasing shock and maskelynitization level (*10*, *43*). The pressure associated with such peak broadening has been calibrated using reverberating shock experiments and assigned to shock pressures of 26 to >45 GPa. However, this spectroscopic shock level barometer, like the maskelynite threshold, likely requires a systematic pressure shift to account for the differences between natural shock loading and reverberation experiments (*43*).

The HP mineral assemblages in many fully maskelynitized shergottites, such as Tissint, Zagami, and Dar al Gani (DaG) 735, are stable at <25 GPa in basaltic bulk compositions (Fig. 1) (*16*). In distinct contrast, these shergottites mostly contain full maskelynite with no birefringent plagioclase, which has been assigned to a pressure of >30 GPa based on previous reverberation shock experiments (Fig. 1). This discrepancy is problematic—if all the shergottites were truly shocked beyond 30 GPa, then postspinel transformation, recrystallization, and local melting would be pervasive. Although complete maskelynitization sets a lower bound of shock pressure in shergottites, more heavily shocked rocks such as Allan Hills (ALH) 77005 and Northwest Africa (NWA) 1950 that reached 35 to 40 GPa (*18*, *44*) commonly contain brown olivine with shock-induced planar deformation features (*36*) and quenched vesicular feldspathic glass instead of maskelynite (Fig. 1B and fig. S10). These textures are reproduced by the extensive deformation and melting (fig. S8) observed in one of our higher-pressure experiments (S1238, 42.4 GPa). The potential HP minerals in these strongly shocked rocks are likely annealed (*25*), resulting from postshock temperature high enough for retrometamorphism, in contrast to the maskelynitized shergottites shocked to <25 GPa. Even for the rare examples of partially maskelynitized shergottites, such as NWA 8159 (Fig. 3) (*17*), the previously determined threshold pressure was still above 25 GPa (Fig. 1). Our single-shock recovery experiments reproduce partial maskelynite textures like those in NWA 8159 (Fig. 3, C and D, and fig. S10) and yield a new partial-to-complete maskelynitization threshold, 17.4 to 21.7 GPa, which is consistent with the majorite-pyroxene assemblage in the same meteorite (Fig. 1) (*17*). On the basis of pressure thresholds, the pressure of <25 GPa inferred from HP phase assemblages of shergottites that reached the onset of postspinel transformation, such as Zagami and Tissint, are therefore sufficient for the observed complete maskelynitization.

The reported LP maskelynitization threshold is also more consistent with untransformed accessory minerals in shergottites, such as baddeleyite (*5*). Zircon and baddeleyite undergo several displacive transitions with low activation energy and fast kinetics (*19*, *20*), allowing transition at low temperature (*45*). Previous maskelynitization barometry indicated shock compression above 30 GPa and 600°C bulk temperature in shergottites (Fig. 1). Such conditions should have caused pervasive transformation of baddeleyite to orthorhombic and tetragonal structures, followed by reversion to polycrystalline monoclinic aggregates upon release, as observed in terrestrial target rocks that experienced similar long-pulse impact events (*20*). The neighborhoods around plagioclase crystals likely experience local temperature even higher than the bulk rock (*27*) due to the compressibility of feldspar and the volume decrease associated with maskelynite formation (Figs. 1A and 2C). Martian baddeleyite can be found entrained in fully maskelynitized feldspar in shergottites and retains magmatic crystallinity, zoning patterns, and U-Pb ages, which has previously been seen as inconsistent with peak shock pressure >30 GPa (*46*). The lower maskelynization pressure, 17.4 to 21.7 GPa, in our study, is compatible with these undisturbed zirconium minerals. Although the mechanism of Pb loss during shock deformation and ZrO_2_ transformation is not fully understood (*47*), moderate values of shock pressure, shock temperature, and postshock temperature, in association with our maskelynitization threshold are more consistent with the observed crystallography and limited resetting of the zircon and baddeleyite. In turn, this result strengthens the case that the <0.6 Ga ages of shergottites are primary crystallization ages and not partially reset values.

### Launch of shergottite from greater depth

Finding agreement between the peak pressures implied by feldspar transformation and those recorded by HP minerals crystallized in melt veins also eliminates the need for complex partial release scenarios featuring an excess pressure spike (for maskelynitization), followed by a stable shock pulse (for HP minerals). Instead, a unified shock pressure of <25 GPa for maskelynite and HP minerals in many shergottites favors models in which the melt veins record plateau conditions at peak pressure lasting 10 to 100 ms (*17*, *33*, *34*). Thus, the whole *P*-*t* profile extracted from analysis of shergottites becomes simple and well-constrained and potentially more suitable for modeling the launch of shergottites from Mars.

Because particle velocity of several kilometers per second corresponds to excessive pressure close to whole rock melting, impact spallation models are proposed for accelerating martian meteorites to escape velocity while limiting the intensity of shock metamorphism that they experience (*6*, *7*). Kurosawa *et al*. (*7*) extracted pressure and velocity histories of multiple tracers in hydrocode spallation simulations and demonstrated that ejection from depth 1 to 2% of the impactor radius is most probable for achieving escape with a plateau-like *P*-*t* profile. Although their modeled impactor of 10-km radius corresponds to a very large crater, the same scaled depth in the case of a smaller impactor would still be consistent with the absolute depth needed to explain the differences noted between martian meteorites and surficial lithologies (*48*) and with the absence of 2π irradiation (*49*). By contrast, in previous scenarios requiring multistage *P*-*t* histories, ejecta likely originate at a depth of <0.5% of the impactor radius. In such models, satisfying the absolute depth, pulse duration, and pressure constraints requires an impactor of several-kilometer radius and a crater diameter of >100 km (*8*). Such a crater size is even greater than the largest candidate craters for the origin of the shergottites, like Mojave (58 km) and Kotka (29 km) (*50*).

Bowling *et al*. (*8*) demonstrate that there is a correlation between impact size and dwell time at a given pressure. Quantitatively, for a peak pressure of ~30 GPa, launched material has a ratio of HP dwell time to impactor radius of up to 20 μs m^−1^, whereas for 20-GPa peak pressure, this ratio decreases to <15 μs m^−1^. Hence, ejection at peak pressure of ~20 GPa rather than 30 GPa implies a 30% larger impactor for the same dwell time (fig. S11). One remaining issue here is that peak pressure may still increase to >30 GPa in material ejected from greater depth but laterally close to the impact center. Elliot *et al*. (*48*) applied a fragmentation model in their simulations and found that a 20-m layer of tuff on top of basalt enhances the overall ejection capability of a 1-km impactor, compared to pure basalt targets, potentially launching material from depth to escape at a lower peak pressure. In summary, somewhat counterintuitively, a decrease in the estimate of peak pressure recorded by the shergottites implies an increase in diameter of the crater from which they were ejected. LP ejection increases the need for special geometries of oblique impact and for mechanisms such as ejecta pileup recognized in recent high-resolution simulations (*7*). In turn, a decrease in the estimate of shock pressure in the shergottites increases the rarity of ejection of unmelted rocks and increases the probability that the known shergottites were ejected by fewer impact events, given their narrow range of cosmic ray exposure ages (*51*). LP maskelynitization combined with HP mineral stability fields indicates that future hydrocode simulations of shergottite launch should focus on a plateau-like *P*-*t* profile and increased depth of origin.

Because maskelynitization of feldspar depends on many variables, including mineral composition, target porosity, and the *P*-*T*-*t*-$\dot{\epsilon }$ path of shock compression, it is important to calibrate shock conditions with suitable experiments. The high deviatoric stress, high strain rate and well-defined shock temperature realized in a one-step shock loading setup are appropriate for interpreting shocked meteorites from impacts on Mars and other basaltic targets like Vesta and the Earth. The methodology of EoS measurement combined with single-shock recovery experiments can be applied to terrestrial basalts, HEDs and lunar rock analogs to better constrain the pressure of the maskelynitized rocks in their corresponding groups.

## MATERIALS AND METHODS

### Martian analog sample

To reproduce the shock conditions in shergottites, we used Saddleback basalt (tables S1 and S2) from the Mojave Desert in southern California (*29*). This nearly holocrystalline basalt, with low porosity (3%) and <10% groundmass, was selected for testing the mechanical design of the Curiosity rover (the Mojave Mars Simulant) because of the similarity of its physical properties to martian rocks (*29*).

### Hugoniot measurement in shock experiments

The Hugoniot EoS of material is most commonly expressed as an empirical linear (or piecewise-linear) relationship *U*_s_ = *C*_o_ + *su*_p_, where *U*_s_ and *u*_p_ are the velocity of the shock front and the particle velocity in the shocked material; *C*_o_ is expected to be the zero-pressure bulk sound speed, and *s* is a dimensionless factor related to the pressure derivative of the bulk modulus. Knowing the *U*_s_-*u*_p_ relationship for each material involved in an experimental impact, one can calculate the shock state pressure and volume (*V*) via the Rankine-Hugoniot conservation equations and the assumption of velocity and stress continuity across interfaces. With additional constraints on the isochoric heat capacity and thermodynamic Grüneisen parameter, the temperature increases across a shock can also be estimated (Fig. 1 and sections S1 and S5) (*25*).

The basalt EoS shots were performed on the 40-mm propellant gun in the California Institute of Technology (Caltech) Lindhurst Laboratory for Experimental Geophysics. More details of experimental setup are in section S1. Shock arrivals at the silvered surface of each mirror generate sharp cutoffs on the Hadland Imacon 790 streak camera image (Fig. 2B). The travel time of the shock wave across the sample is determined by the average of the time differences between the light cutoffs at the two sample flat mirrors and at the adjacent driver plate mirrors (*t*_1_ and *t*_0_ in Fig. 2B), converted to travel time and then shock wave speed (*U*_s_). The impedance match solution (which imposes continuity of particle velocity and normal stress at the driver-sample interface), the known Hugoniot of the driver, and the measured *U*_s_ yield the sample particle velocity *u*_p_.

Assuming the preshock velocity of the sample is zero, the Rankine-Hugoniot equations for conservation of mass, momentum, and energy (*E*) across the shock front can be written as

$$\rho_{o}\;U_{s}=\rho \;(U_{s}-u_{p})$$ (1a)

$$(P-P_{o})=\rho_{o}\;U_{s}\;u_{p}$$ (1b)

$$E-E_{o}=(P+P_{o})(V-V_{o})/2$$ (1c)

These equations allow calculation of *P*, ρ, and (*E* − *E*_o_) in the shock state given measurements of *U*_s_, *u*_p_, and initial density ρ_o_. In the case of a two-wave structure, the HP slow wave also follows the impedance match at driver-sample interface.

When the target material undergoes an elastic-plastic or low-density to high-density transition, a two-wave structure forms, whereby the slower second wave has a higher pressure. In this case, only the arrival of the faster first wave can be captured by the streak camera, but the impedance match at the driver-sample interface needs the *P*, *U*_s_, and *u*_p_ of the second wave. For such cases, we measure the slow wave and fast wave(s) by adding an inclined mirror behind the sample at an angle ϕ, in addition to the regular flat mirrors on the driver and basalt sample disc (Fig. 2A). The inclined mirror is wedge-shaped so that its refraction and reflection offsets cancel out and light is reflected back to the center of the camera sensor. When a shock wave arrives at the rear free surface of the sample, decompressed material traverses the vacuum at free surface velocity *U*_fs_ and hits the inclined mirror, creating an oblique cutoff (γ in Fig. 2B) along the inclined mirror streak. *U*_fs_ is determined from the corresponding angle γ on the streak cutoff using

$$U_{fs}=(W\;\cdot \tan\phi )/(m\;\cdot \tan\gamma )$$ (2)

where *W* and *m* are the writing rate and magnification of the streak image, respectively (*37*). Thus, the arrival of the second wave, with higher *u*_p_ and *U*_fs_ than the first wave, is shown by the change in γ angle on the streak cutoff (*t*_2_ in Fig. 2B). Assuming the release adiabat is exactly a reflected Hugoniot, *U*_fs_ is expected to be 2*u_p_ (section S1). The timing of the slope transition also approximately indicates the second wave velocity by

$$U_{s2}=[d+(t_{2}-t_{1})*U_{fs1}]/(t_{2}-t_{0})$$ (3)

where *d* is the sample thickness, and the meaning of each time *t*_i_ is indicated on Fig. 2B. In the case that *U*_fs_ ≠ 2*u*_p_, which is common for a wave in a transformed phase, the final particle velocity can be calculated by iteration between *U*_s2_ and the impedance match solution at the sample-driver interface (fig. S1).

In our first two EoS shots (1123 and 1124), we observed *U*_s_ decreasing from 6136 to 5232 m s^−1^ as flyer velocity *U*_fp_ increases from 1394 to 1598 m s^−1^ (fig. S2), indicating that, in this range, the sample undergoes a density transition to a higher density state that produces a two-wave structure; the slower, higher-pressure wave is not captured by the flat mirror streak cutoffs.

To determine *U*_s_ and *u*_p_ for both the first and second waves, the third shot (1126), fired at an intermediate *U*_fp_ of 1479 m s^−1^, used an inclined mirror method to measure multiple wave velocities. The first wave has *U*_s1_ = 5766 m s^−1^ and *u*_p1_ = 1009 m s^−1^, whereas the second and final waves have *U*_s2_ = 5232 m s^−1^ and *u*_p2_ = 1297 m s^−1^. Interpreting this two-wave structure as recording a density transition, we find that the density transition is complete at 16.6 GPa (much higher than the HEL, which can generate a similar two-wave structure in weaker shocks). The data from all three EoS shots can be summarized by a piecewise-linear fit, with negative slope *U*_s_ = 9594 − 3.72*u*_p_ for *u*_p_ between 900 and 1200 m s^−1^ and a high-density phase at *u*_p_ ≥ 1200 m s^−1^ characterized by *U*_s_ = 3603 + 1.256*u*_p_. The transition pressure interval extends from 15.4 to 16.6 GPa and results from incomplete density transition and mixing of low- and high-density states along the Hugoniot. The EoS shots are summarized in fig. S2 and table S3. These fits are sufficient to enable calculation of pressures in our recovery experiments. The LP Hugoniot is estimated (Fig. 2C) using the zero-pressure sound speed of Saddleback basalt (*29*) and does not affect the pressure calculation of recovery experiments (fig. S2). Comparison among Hugoniots from Saddleback basalt and other feldspar rocks is discussed in the section S2.

### Shock recovery experiments

Discs of Saddleback basalt with a diameter of 5 or 7.6 mm and thickness *d*_sample_ of 1.0 to 5.0 mm were used for total of seven shock recovery experiments. The samples were embedded in 304 stainless steel (SS304) chambers and affected by tantalum flyer plates of thickness *d*_flyer_ from 1.5 to 2.1 mm (table S3 and section S3). In practice, a thickness ratio *d*_sample_/*d*_flyer_ of <1 allows enough shock transits across the sample and chamber to effectively achieve an ultimate peak pressure by reverberation, before the rarefaction wave from the back of the flyer arrives. In four of the total seven recovery shots, the sample experienced full reverberation to peak pressures from 33 to 42 GPa after first shock pressures from 16 to 19 GPa (table S3 and fig. S5). Three more recovery shots with similar impact velocities and *d*_sample_/*d*_flyer_ of >2 were performed. In this geometry, wave propagation calculations show that the front portion of the sample is shocked only once before release to the same range of initial pressures, 16 to 19 GPa. In each of these three nonreverberating experiments, some part of the sample near the steel back wall also experienced one reflected shock (Fig. 3 and figs. S5 to S8).

The affected target assemblies were cut parallel to the impact direction into two equal halves. One half was polished into a thick section for reflected light imaging. The other half was sliced and mounted in Petropoxy 154 to make a standard 30-μm thin section for examination in cross-polarized transmitted light. Both thick and thin sections were analyzed with a Zeiss 1550VP field-emission scanning electron microscope in the Division of Geological and Planetary Sciences at Caltech. Backscattered and secondary electron images are used to observe the microtextures of the shocked basalt. Energy-dispersive x-ray spectroscopy with an Oxford X-Max silicon drift detector was used to measure the chemical composition at 15-kV accelerating voltage and 4- to 6-nA beam current, achieving more than 200 counts per channel and 40% dead time. We also used EBSD to investigate the crystallinity of shocked minerals. Regions with resolved diffraction bands were indexed with mean angular deviation values less than 0.8° (fig. S7). Regions showing no resolvable diffraction bands are considered amorphous. The band contrast metric quantifies the degree of crystallinity at the EBSD point analysis. Quantitative chemical analyses of rock-forming minerals and glasses were performed using a JEOL JXA-8200 electron microprobe (Wavelength-dispersive x-ray spectroscopy: 15 kV; 5 to 10 nA; beam in focused mode) interfaced with the probe for Electron Probe Micro-Analysis program from Probe Software Inc. Standards for these analyses were synthetic fayalite (Si*K*α for olivine, Fe*K*α), Shankland forsterite (Mg*K*α), synthetic Mn_2_SiO_4_ (Mn*K*α), synthetic anorthite (Al*K*α, Si*K*α for feldspar, Ca*K*α), Amelia albite (Na*K*α), Asbestos microcline (K*K*α), synthetic TiO_2_ (Ti*K*α), and synthetic Cr_2_O_3_ (Cr*K*α). Quantitative elemental microanalyses were processed with the CITZAF correction procedure (table S1).

Because our impedance match calculations assume one-dimensional flow, we focused on the central portion of the sample when determining the abundance of completely or partially amorphized domains to avoid regions affected by possible edge effects and rock-metal friction (fig. S6). Although additional amorphous material commonly occurs along large fractures and at the capsule edges (Fig. 3A and fig. S6), these are considered to be associated with edge conditions and are not considered when defining thresholds for maskelynite formation. The difference between maskelynitization thresholds obtained by shocking Saddleback basalt with those from other compression techniques is discussed in section S4.

## Supplementary Materials

### This PDF file includes:

Supplementary Text Sections S1 to S6 Figs. S1 to S11 Tables S1 to S4 References
