# Supplementary material for: Shock-recovered maskelynite indicates low-pressure ejection of shergottites from Mars
Source: Sci Adv. 2023 May 3;9(18):eadf2906. doi: 10.1126/sciadv.adf2906 (PMC10156110; doi:10.1126/sciadv.adf2906)
Supplement: Supplementary file 1 — Supplementary Text Sections S1 to S6 Figs. S1 to S11 Tables S1 to S4 References [file sciadv.adf2906_sm.pdf]

Science Advances
AAAS

Supplementary Materials for

Shock-recovered maskelynite indicates low-pressure ejection of shergottites from Mars

Jinping Hu *et al.*

Corresponding author: Jinping Hu, jinping@caltech.edu

Sci. Adv. **9**, eadf2906 (2023)
DOI: 10.1126/sciadv.adf2906

This PDF file includes:

Supplementary Text
Sections S1 to S6
Figs. S1 to S11
Tables S1 to S4
References

Supplementary Text

S1. Hugoniot equation of state measurement of Saddleback basalt

The basalt EoS shots were performed on the 40 mm propellant gun in the Caltech Lindhurst Laboratory for Experimental Geophysics. The flyer velocity U_{fp} of the projectile before impact is measured by the interruption of three lasers that cross the flight path; it is considered accurate and precise to 0.1%. The shock transit through the sample is recorded with a Hadland Imacon 790 streak camera fitted with a digital image capture system. Light from a long-pulse Xenon flash lamp is reflected by the array of rear-surface target mirrors onto the focal plane of the camera, which is set to record 2-4 μs streak durations. With 4008 pixels in the streak direction, this provides a nominal time resolution of 0.5-1 ns. The mapping from pixel number on the image to time is calibrated with a quadratic fit to the signal from an 82.800 MHz radio oscillator, offering first-order correction for variations in streak rate along the streak. Shock arrivals at the silvered surface of each mirror generate sharp cutoffs on the streak-camera image (Fig. 2b in the main text). The travel time of the shock wave across the sample is determined by the average of the time differences between the light cutoffs at the two sample flat mirrors and at the adjacent driver plate mirrors (t_1-t_0 in Fig. 2b in the main text). Then the pre-measured sample thickness is used to convert travel time to shock-wave speed (U_s). The impedance match solution (which imposes continuity of particle velocity and normal stress at the driver-sample interface), the known Hugoniot of the driver, and the measured Us yield the sample particle velocity u_p (Fig. S1 and S2).

As stated in Methods of the main text, assuming the pre-shock velocity of the sample is zero, the Rankine-Hugoniot equations for conservation of mass, momentum and energy (E) across the shock front can be written as:

$$\rho_o U_s = \rho (U_s - u_p) \qquad (1a)$$

$$(P - P_o) = \rho_o U_s u_p \qquad (1b)$$

$$E - E_o = (P + P_o)(V - V_o)/2 \qquad (1c)$$

These equations allow calculation of P, ρ, and $(E - E_o)$ in the shock state given measurements of shock velocity U_s, particle velocity u_p, and ρ_o. In the case of a two-wave structure, the high-pressure slow wave also follows the impedance match at driver-sample interface (see Methods in the main text). Results for the EoS are summarized in Table S3.

S2. Shock Hugoniot equation of state in plagioclase-bearing rocks

Our recovery experiments targeting this pressure range show that this is the same pressure as the plagioclase-maskelynite transition upon single-shock loading (Fig. 1-2 in the main text), strongly suggesting that densification of plagioclase enabled by amorphization is the cause of the observed increase in density on the Hugoniot. We note that it is quite challenging to observe whether this feature is a general property of the Hugoniot of plagioclase-rich rocks, given the sparsity of the data in the appropriate pressure range (Fig. S2 and S4).

Limited Hugoniot data are available for other basalts such as Vacaville (*37*) and Kinosaki (*30, 31*); both these materials have significant fractions of fine groundmass. The Hugoniot for Vacaville basalt shows a clear two-wave structure due to elastic-plastic transition with a Hugoniot elastic limit at ~5 GPa (*37*). The two-wave structure observed in Kinosaki and Saddleback basalts above 15 GPa is unlikely to relate to the elastic-plastic transition.

Experiments on Kinosaki basalt used piezoresistive gauges to directly measure the pressure-time profile of the sample (*30*). The data also show low- and high-density regimes and a mixed regime of near flat reported U_s-u_p correlation for u_p between 500 and 1200 m s^{-1} (Fig. S2).

The location of this mixed regime, also confirmed by the slow rise in pressure profile (Fig. S3), is in good agreement with our Saddleback basalt results, but the detailed shape of the U_s-u_p relations appears quite different. We note, however, that the authors originally determined the shock-wave velocity by manually picking free-surface arrivals and onset of rarefaction waves in their wave profile data. For the mixed regime, the pressure profile is complex and the rarefaction-wave arrival is not obvious (Fig. S2). To reduce the possible uncertainty from subjective selection, we take advantage of the first order measurement of shock pressure by the piezoresistive gauges and recalculate the corresponding U_s-u_p by impedance match with the driver. The recalculated Hugoniot indeed shows a clear negative slope for u_p between 500 and 1200 m/s and the Hugoniot slope in the high-density regime matches well with our data (Fig. S2a). Despite the grain-size difference of the two basalts, there is a similar phase transition at around 13-18 GPa. The Hugoniot data for other plagioclase-bearing rocks are mostly not focused on this window of pressure and data density is insufficient to observe a phase transition (Fig. S4).

Ahrens et al. (*37*, *55*) measured the Hugoniot of several feldspars and observed a phase change to a high-pressure phase, presumably tetragonal polymorph, above 40 GPa with a mixed regime between 20-40 GPa. There is at most one point in the 15-20 GPa window for each composition and therefore these data do not test for the occurrence of another phase transition in this range. The release behavior of pure feldspar from above 20 GPa, showing $U_{fs} < 2u_p$ (*55*), also contrasts with the release behavior shown by basaltic rock (*37*) (Fig. S1 and Table S3).

In summary, both Saddleback basalt and Kinosaki basalt display a phase transition at 15-16 GPa with a significant density increase that is not resolvable in data on pure plagioclase. Nevertheless, the coincidence of this pressure with the onset of the plagioclase to maskelynite transition in our recovery experiments strongly suggests that it is the amorphization of the large volume fraction of plagioclase in these basalts that is responsible for the abrupt increase in density along the single-shock Hugoniot.

S3. Shock recovery experiments of Saddleback basalt and maskelynitization

Discs of Saddleback basalt with a diameter of 5 or 7.6 mm and thickness d_{sample} of 1.0 to 5.0 mm were used for total of 7 shock recovery experiments. The samples were embedded in 304 stainless steel (SS304) chambers of dimensions previously shown to retain momentum and return intact samples. The target assemblies were impacted by Ta flyer plates of thickness d_{flyer} from 1.5 to 2.1 mm (Table S3). The pressure in the SS304 chamber (obtained by impedance match between Ta and SS304) after the impact is the highest possible pressure in the target assembly, because basalt has a lower shock impedance than steel (Fig. S1). When the shock wave enters the sample from the driver, impedance match requires that the pressure of the driver decrease to reach continuity with the sample; we assume that the driver releases along its reflected Hugoniot. Subsequently, after the shock wave traverses the sample and reaches the back wall (again, SS304 of high impedance), a reflected shock occurs in the sample and reaches a higher mutual pressure with the back wall. In reverberation experiments, enough such reflections occur to approximate sample-chamber equilibration at the original shock pressure in the capsule material, which is independent of the EoS of the sample and of the loading path (Fig. S5).

We investigated recovered samples of three non-reverberating shots and four reverberation shots (Table S4), with petrographic microscope (see Fig. 3 in the main text), electron microscope and electron back-scatter diffraction (Fig. S6-S8).

The pressure-time profile of each shot was modeled using the WONDY 1-D hydrocode (*56*), to verify the interpretation that the two regions observed in non-reverberating recovered

samples are in fact single-shock and double-shock zones. The ***P-t*** profiles indicate that for a thick sample (e.g. S1240), the front part experiences a single shock for ~1 μs before a rarefaction wave originating from the back of the flyer plate arrives. Moving through the single-shock zone from sample front towards back, the duration of the high-pressure pulse decreases. The back part experiences a first shock and a reflected shock from the back wall before arrival of the rarefaction wave. These profiles, with two zones of distinctly different peak pressure, are consistent with the two-zone maskelynitization found in the recovered samples (Fig. S6-7). For a thin sample with full reverberation (e.g. S1238), the rarefaction wave traverses the sample and releases the pressure more quickly, resulting in a shorter pulse overall.

The crystallinity of feldspar in shock-recovered basalt is verified by both optical isotropy and electron backscattered diffraction. The comparison of two techniques suggests that EBSD is most sensitive to crystallinity in grains with ultra-low optical birefringence (Fig. S7). In general, all shocked plagioclase shows decreased birefringence and poorly-defined twin planes, including the single-shock zone (15.8 GPa peak pressure) in S1240 (Fig. 3 in main text and Fig. S8).

S4. Maskelynitization in reverberating shocks and other compression experiments

Laboratory shock experiments achieve one-step loading achieve pulse duration, strain rate, deviatoric stress and pressure-temperature correlation most similar to martian impacts and therefore are the most suitable for building a thermobarometer from maskelynitization. However, static and rapid compression studies provide useful insights into the effects of temperature, duration, and strain. Here we will discuss these factors in our experiments and demonstrate that one-step-loading propellant experiments have several major advantages over reverberation or laser techniques that support the conclusion that maskelynite formation in martian meteorites occurs at pressures comparable to those indicated by other shock features.

Reverberation experiments have been performed on both feldspar single grains and plagioclase-bearing rocks. For An_{50-80} plagioclase, relevant to shergottites, experiments on randomly oriented labradorite An_{63} single crystals showed onset of maskelynitization above 28.7 GPa and full amorphization above 30 GPa (*12*) (Fig. 1 in the main text). Similarly, An_{65} labradorite single crystals impacted along the a-axis and anorthosite with polycrystalline An_{77} bytownite display partial and complete maskelynitization at 26.5 GPa and 28-30 GPa, respectively (*13*) (Fig. 1 in the main text).

Besides single crystals and monomineralic rocks, more realistic samples of the basaltic target rock from Lonar Crater, India, were extensively studied for comparison to terrestrial and lunar impact rocks (*11*). The starting materials, with up to 15% An_{68-69} labradorite phenocrysts, were from a deep drill core; they are nonporous and were not affected by shock metamorphism from cratering. In reverberating recovery experiments, the partial maskelynitization begins at 25 GPa and is complete at pressure greater than 33 GPa (Fig. 1 in the main text). Unexpectedly, the pressure of complete transition is higher than that of labradorite single crystal, whereas partial transition begins at lower pressure in basalt. The opposite might be expected because of the local heating from grain-boundary friction during shock. Nevertheless, each of the reverberation experiments show much higher maskelynitization pressures than our Saddleback basalt subjected to one-step loading (Fig. 1 in main text). This pressure discrepancy indicates the significance of shock temperature, pulse duration, and shear stress that we will discuss in the following paragraphs.

To explore the effects of shock temperature, we present the maskelynitization threshold in pressure-temperature (***P-T***) space for recovery samples (Fig. 1 in main text; see section S4 for

details of temperature calculations). The *P-T* curves indicate that the shock temperature variation for single crystal versus basalt samples and for one-step versus reverberation loading are not significant for the maskelynite transitional regime of 15-25 GPa (Fig. 1 in main text). In reverberation experiments, labradorite achieves 50-100 K higher overall shock temperature than dense basalt, which may explain the lower pressure for complete maskelynitization in labradorite. However, the pressure of onset of partial maskelynitization is not always negatively correlated with the temperature difference. Even though the shock temperature is higher for the more compressible labradorite rock, the frictional heating and shear stress concentrated at grain boundaries in basalt samples likely enhances partial maskelynitization locally. Similarly, the comparison with one-step-loading on porous Saddleback basalt also indicates that temperature is likely not the primary source of differences between our results and previous reverberation studies. Even though our experiments on Saddleback samples beyond 30 GPa achieve shock temperatures 200 K higher than reverberation to equal pressure, maskelynitization for Saddleback basalt is completed at much lower pressures of 15-25 GPa (Fig. 1 in main text). In this range, the temperature is almost identical to reverberation of dense rocks.

The effects of temperature at pressures <25 GPa can be interrogated in more detail with available pre-heated shock recovery and static compression experiments. Takenouchi et al. (*36*) pre-heated olivine-phyric basalt to 1073 K in an attempt to compensate for the artificially small temperature deficiency that result from shock compression by reverberation. Even with this significantly elevated temperature, the onset of partial maskelynitization was only observed at >22.2 GPa. This threshold pressure is ~4 GPa lower than cold-start reverberation shots but still 5 GPa higher than our single-shock loading experiments. Similarly, Huffman et al. (*32*) carried out reverberating shock recovery on cold and pre-heated (~1073 K) granite and found maskelynitization thresholds of 23 GPa cold and 16 GPa preheated. Granite has a lower overall shock impedance than basalt and therefore the pressure decrease of maskelynitization caused by high shock temperature might be more significant. The *P-T* phase boundary for maskelynite transition in hydrostatic compression was determined precisely by Kubo et al. (*21*), using initial cold compression followed by isobaric heating with real-time *in* situ X-ray diffraction monitoring to identify the point of complete amorphization. The determined line is therefore closer to a thermodynamically (meta)stable phase boundary than any shock recovery result (Fig. 1 in main text). The "Clapeyron"slope is –0.01 GPa/K, i.e. a 400 K temperature increase lowers the threshold pressure for complete amorphization by 4 GPa. This result is in general agreement with the difference in maskelynitization pressures between pre-heated and room-temperature reverberation experiments. Unsurprisingly, static compression also indicates that the duration of exposure to peak pressure affects the effective transition threshold, which is another difference between propellant single-shock, reverberation loading and laser shock. For a reverberation experiment of > 30 GPa peak pressure, the peak pressure typically lasts for < 0.5 μs and the sample spends most of total duration of the shock event (~1200 ns in typical chamber designs) at or below 15 GPa (Fig. S3). The laser shock, although achieving peak pressure in one shock, has duration on the order of 10 ns (*24*). Although it is currently impossible for laboratory shocks to achieve pulse durations >1 ms that match planetary impacts (*33*), the peak pressure in our single-shock loading experiments holds for >3 μs and better approximates conditions near the central impact sites in nature (*25*).

Strain rate and deviatoric stress in the sample are also significant quantities influencing the amorphization of feldspar, perhaps even the principal driving factor for amorphization (*22*, *32*). Sims et al. (*23*) quantitatively investigated the amorphization strain-rate with rapid

compression experiments and ***in situ*** time-resolved diffraction. For anorthite, the onset of maskelynitization occurs below 11.5 GPa with compression rates of 0.1-1 GPa s^{-1} (presumed to correspond to strain rates of ~10^{-3} to 10^{-1} s^{-1}) and complete amorphization pressure varied with changing compression rate from 21 to 32 GPa, in a non-monotonic way. In contrast, the strain rate behind natural and laboratory shock front is ~10^{5} s^{-1} in this pressure range, decaying to at least 10^{0} s^{-1} far from the impact center (*40*). Nevertheless, the onset pressure is still much lower than the threshold of >20 GPa determined by reverberation experiments on anorthite-bearing (An_{94}) gabbro (*15*). At > 18 GPa s^{-1} compression rate, maskelynitization appears to occur abruptly and completely at <12.5 GPa without a partial transition interval, which is comparable to static compression results (*21*). So the onset of maskelynitization in static and rapid compression with strain rates of 10^{-5} to 10^{-1} s^{-1} (10-15 GPa) is much lower than observed in shock reverberation experiments with strain rates ~10^{5} s^{-1}, even though increasing strain rate appears to promote lower threshold pressures. It is likely that the effects of pressure pulse duration act to offset the strain-rate effect in rapid compression and reverberation recovery experiments.

The deviatoric stress environment created by single-shock loading is likely the most critical difference from reverberation loading. In a reverberating loading path, most of the compression is accomplished by reflected shocks transiting material that has already exceeded its elastic limit and failed, losing strength (Fig. S1). Although the mechanics of material healing and re-acquisition of shear strength after shock yielding are not well understood, it is very likely (if only because each shock is transiting successively hotter and hence weaker material) that the overall shear stress achieved is much less than in a single-step shock even to a lower peak pressure. We suggest that this is the key aspect in which our experiments better resemble the $\boldsymbol{P\text{-}T\text{-}t\text{-}\dot{\epsilon}}$ and deviatoric stress conditions experienced by martian basalts upon impact and ejection.

In our shock-recovery experiments, partial or full-grain amorphization does occur along large fractures and adjacent to basalt-steel boundary even at 17.4 GPa or below (Fig. S6). We do not consider these features in determining the pressure threshold for maskelynite because displacive fractures and high-impedance material like metal is rare in crystalline plagioclase-bearing shergottites. In other words, for best comparison to shergottites, we identify the pressure threshold for Saddleback basalt based on maskelynite occurrence in the coherent bulk part of the sample.

Compared to our single-shock experiments, a planetary impact provides longer duration (*33*), more turbulent flow (*41*), and extensive shear during excavation (*42*). All these effects would only further lower the pressure threshold for maskelynite. Hence, we argue that the pressures of partial and full maskelynitization of feldspar in our experiments provide robust upper limits for the corresponding pressure thresholds in shergottites.

S5. Calculation of shock temperatures

Details of the thermodynamics and equation derivation in this method are discussed in Hu and Sharp (*25*). Here we provide the equations directly needed for the shock temperature calculations. The thermodynamic properties are listed in Table S4. The estimated shock temperatures due to one-step shock loading of Saddleback basalt along the Rayleigh line (Fig. S9) are calculated by solving the equation that equates changes in the state variable internal energy along two different paths from the initial state to the Hugoniot state:

$$E_h = E_s + E_{tr} + E_v, \qquad (4)$$

where E_h is the internal energy increase from the initial state at volume V_o to a Hugoniot point at compressed volume V, E_s is the energy increase between these two volumes along the reference isentrope, E_{tr} is the enthalpy difference between crystalline plagioclase and glass at the reference pressure, and E_V is the energy increase due to isochoric heating from the isentrope to the Hugoniot at compressed volume V.

The E_h of one-step loading is calculated directly from Rankine-Hugoniot equation

$$E_h = E - E_o = (P + P_o)(V - V_{oo})/2 \qquad (5)$$

where V_{oo} is the bulk specific volume of the starting material, averaged over the scale of observation, which may encompass some porosity. The reference isentrope is expressed by a 3rd order Birch-Murnaghan EoS

$$P_S = 3K_{oS}\, f(1+2f)^{\frac{5}{2}}\,(1+\tfrac{3}{2}(K_S'-4)\,f) \qquad (6)$$

where K_{oS} is the zero-pressure isentropic bulk modulus, K'_S is the pressure derivative of the bulk modulus and f is the Eulerian finite strain parameter

$$f = \tfrac{1}{2}[(\tfrac{V_o}{V})^{\frac{2}{3}} - 1]. \qquad (7)$$

This expression can be integrated to obtain E_s

$$E_s = \int_{V_o}^{V} P_s dV. \qquad (8)$$

V_o and K_{oS} need to be the zero-pressure specific volume and bulk modulus of the material in the condensed state (Fig. S9); for porous or transformed samples, V_o may be smaller than V_{oo}. In the limit of zero strain, K_{oS} and K'_S are related to the coefficient of the linear Hugoniot $U_s = C_o + s\, u_p$ by

$$K_{oS} = C_o^2\, \rho_o \qquad (9)$$

$$K'_S = 4s - 1. \qquad (10)$$

The temperature T_s on the isentrope is obtained from a model of the volume-dependent Grüneisen parameter $(\gamma/\gamma_o) = (V/V_o)^q$:

$$T_S = T_o\, e^{\frac{\gamma_o}{q}[1-\left(\frac{V}{V_o}\right)^q]} \qquad (11)$$

where $q = 1$ for most solids and γ_0 is the Grüneisen parameter of the material at V_o.

E_{tr} is the zero-pressure enthalpy change of the labradorite to maskelynite transition, taken from Navrotsky (*57*). E_v is given by the Mie-Grüneisen EoS,

$$E_V = E_H(V) - E_S(V) = \frac{V}{\gamma(V)}(P_H(V) - P_S(V)) \qquad (12)$$

and therefore the final temperature is

$$T_H = T_S + T_V = T_S + \frac{E_V(V)}{C_V} \qquad (13)$$

where C_v is the isochoric heat capacity.

All the thermodynamic parameters used to calculate the shock temperature are listed in Table S4. It is worth noting that each shock temperature on the Hugoniot is determined using the isentrope as a reference and is independent of other Hugoniot points. This allows us to focus on the shock temperatures of the transition regime based on local measurements of the Hugoniot (Fig. 1 the in the main text), without the need to determine the Hugoniot of the low-pressure regime. For comparison, we also calculated the temperatures for reverberation experiments using the same method (Fig. 1 the main text). The only difference is that reverberation results in a multi-step loading whereby the energy on the Hugoniot includes work from every re-shock, meaning a series of trapezoids under the loading path in *P-V* space.

One more complication arises with reflected shocks and phase transition, where state points in the *P-V* plane cannot be calculated directly from the standard Rankine-Hugoniot

equations and U_s-u_p pairs. We again use the Grüneisen model to calculate the pressure difference between initial and transformed or reflected states at a given volume, by satisfying the energy difference assuming a Mie-Grüneisen EoS (*25*). For a reflected shock:

$$P_2 = [P_H - \rho_2\gamma_2(P_H - P_1)(V_o - V_2)/2]/[1 - \rho_2\gamma_2(V_1 - V_2)/2] \quad (14)$$

where (P_1,V_1) and (P_2,V_2) are states before and after passage of the reflected shock and P_H is the nominal pressure assuming a one-step shock compression from V_o to V_2.

Similarly, for a phase transition:

$$P_c = [P_b\,(1- \rho\gamma(V_{ob} - \underline{V})/2) + \rho\gamma\ \Delta E_{tr}]/[1 - \rho\gamma(V_{oa} - V)/2] \quad (15)$$

where (P_c,V) are for the transformed phase, ΔE_{tr} is the zero-pressure transformation enthalpy, V_{oa} and V_{ob} are (nominal) zero-pressure volume of the initial and transformed phases, and P_b is the nominal pressure of the initial phase at V.

S6. Implications for other shocked meteorites.

Similar to shergottites, a likely decrease of 5-10 GPa in the thresholds for partial and full maskelynitization of anorthite in eucrites, compared to estimates based on shock-reverberation experiments, can also yield better consistency with peak pressures inferred from their HP mineral assemblages (*16*) and decrease the impact velocities on 4 Vesta to avoid a catastrophic impact. The shocked ordinary chondrites, in contrast, experienced much longer shock pulse of seconds. In that case, the long duration is the key factor that regular shock experiments cannot simulate. Static or rapid multi-anvil compression can provide useful maskelynitization threshold for interpreting the shocked albite in ordinary chondrites (*23*).

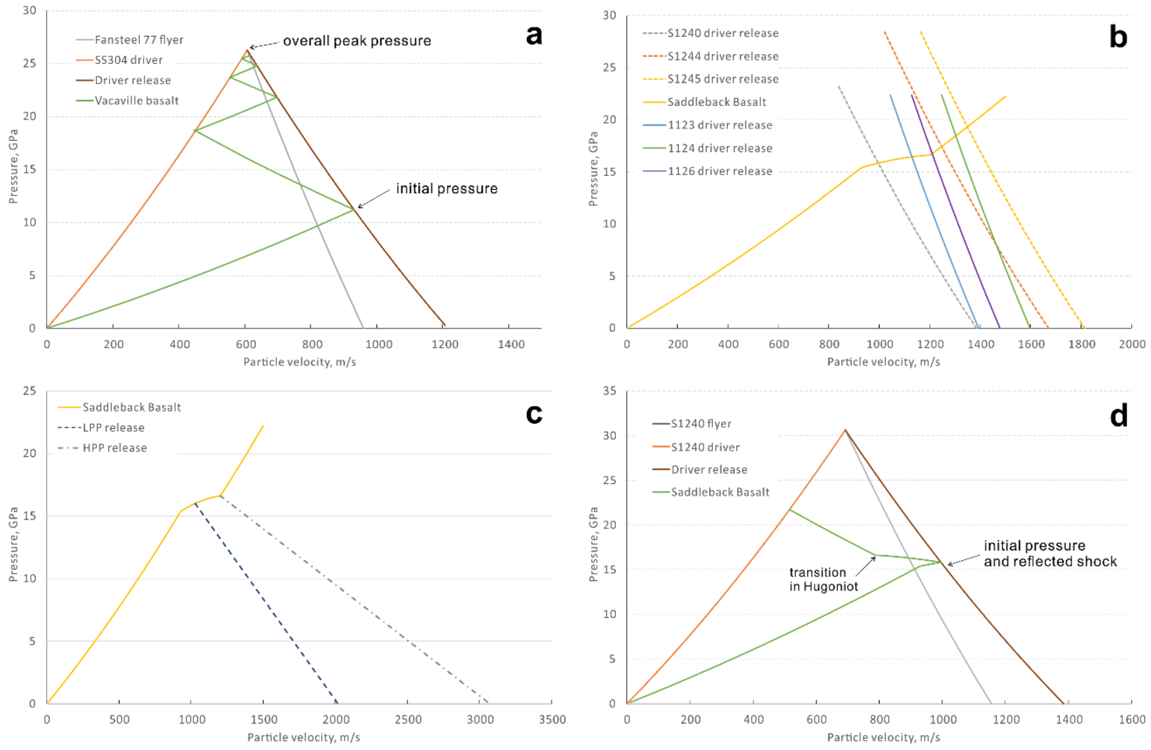

Fig. S1. Shock impedance match and release paths of basalt. (a) full reverberation of dense Lonar basalt in Kieffer et al. (*11*), in which 25 GPa peak pressure did not enable maskelynitization. The Hugoniot is estimated by data on Vacaville basalt in Ahrens and Gregson (*37*). (b) Shock impedance match for EoS (1123, 1124, 1126; solid lines for Ta driver releases) and recovery shots (S1240, S1244, S1245; dash lines for SS304 driver releases) of Saddleback basalt. The intersection between the basalt Hugoniot and the driver release path determines the shock state before any possible reflection. (c) Release path of low-pressure (LPP) and high-pressure (HPP) phases of Saddleback basalt, indicated by free surface velocity at sample rear. (d) Impedance match and shock reflection of shot S1240. Maskelynitization occurs in the reshocked region of recovered sample.

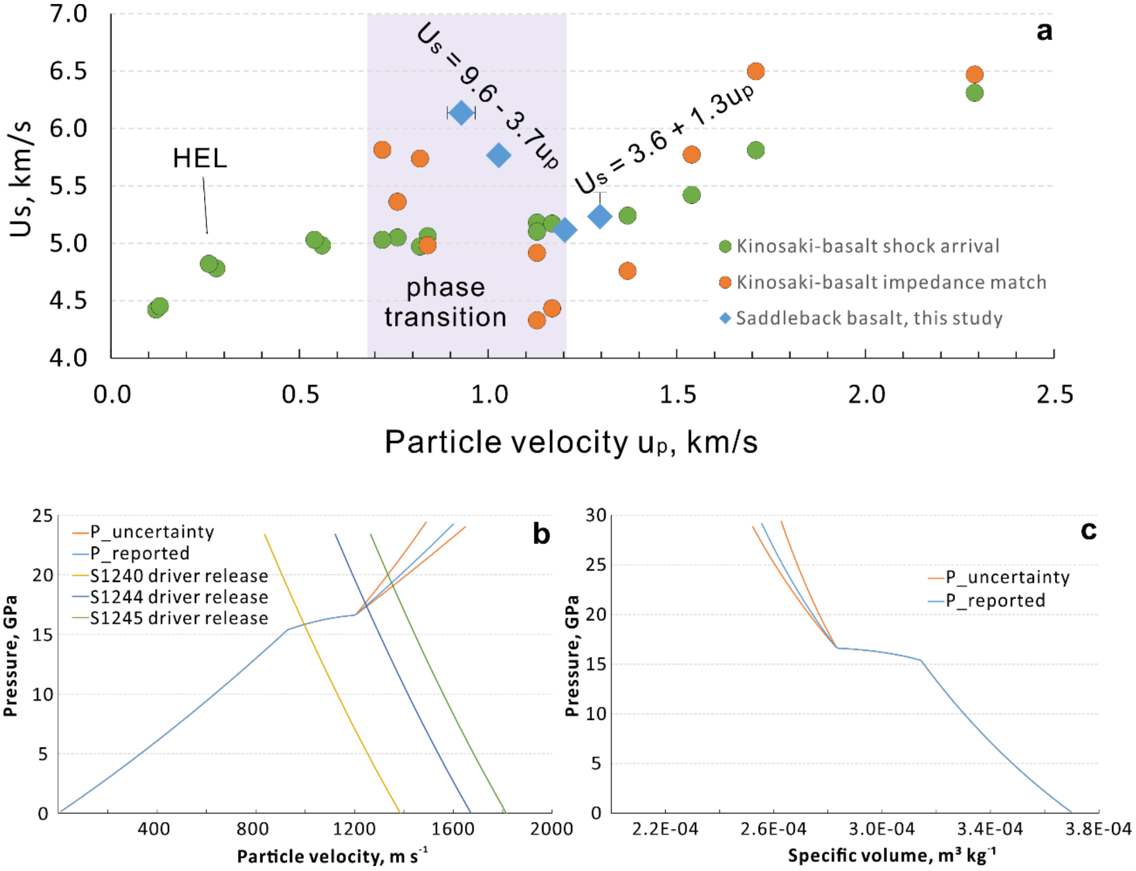

Fig. S2. Shock-wave velocity (U_s) versus particle velocity (u_p) Hugoniot (a), recovery impedance match (b) and *P-V* Hugoniot (c) of Saddleback basalt, with the range of uncertainty in the Hugoniot. (a) Blue diamonds are measurements of Saddleback basalt. The change in slope indicates a phase transition. Linear equations are given for the U_s-u_p Hugoniots of the mixed and high-density regimes. Error bars smaller than the symbol are not shown; asymmetrical uncertainty results from the combination of sample heterogeneity and tilted shock front; corresponding uncertainty in pressure and volume is shown in (b-c). The reported Hugoniot of Kinosaki basalt (*30*) (green circles) is recalculated using the impedance match method (orange circles) to correctly account for two-wave structures, yielding a phase transition similar to that observed in Saddleback basalt (see section S2 and Fig. S3). The main source of uncertainty is a tilted shock front in shot 1124 (U_{fp} = 1.598 km/s). The uncertainty in the Hugoniot translates into a ±1.5 GPa uncertainty on pressures for recovery shots in the pressure range of interest.

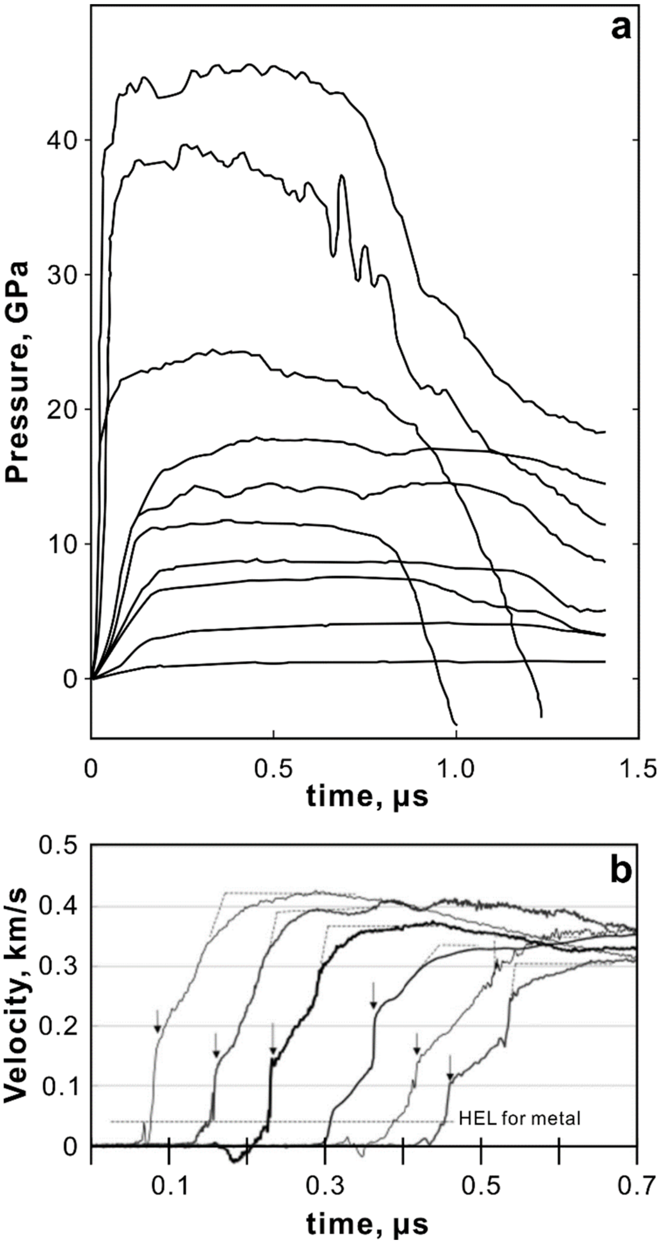

Fig. S3. Pressure and velocity profile of shocked Kinosaki basalt. a) Piezoresistive gauge pressure profile. Experiments with two-wave structures due to elastic-plastic transition or phase transition exhibit slow apparent shock rise times. The arrival of rarefaction wave is difficult to determine for shots < 15 GPa, after Nakazawa et al. (*30*). b) VISAR velocity profiles up to 12.2 GPa. Arrows mark the arrive of elastic wave. The pure plastic waves still show complex slow rising of pressure, after Sekine et al. (*31*).

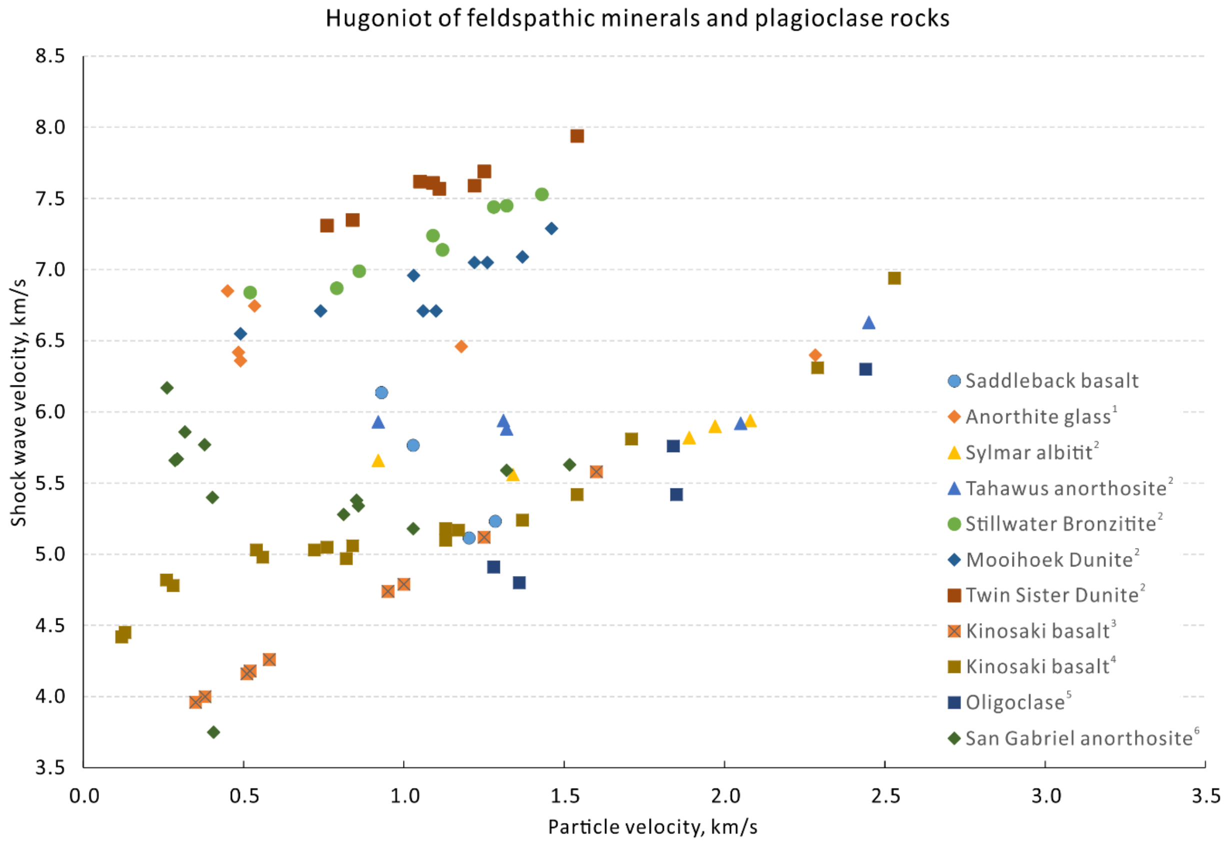

Fig. S4. Hugoniot data for plagiclase rocks, feldspathic minerals and glasses plus one dunite in the U_s-u_p space. References: [1]Boslough et al. (*58*); [2]McQueen et al. (*59*); [3]Sekine et al. (*31*); [4]Nakazawa et al. (*30*); [5]Ahrens et al. (*55*); [6]Ahrens and Gregson (*37*).

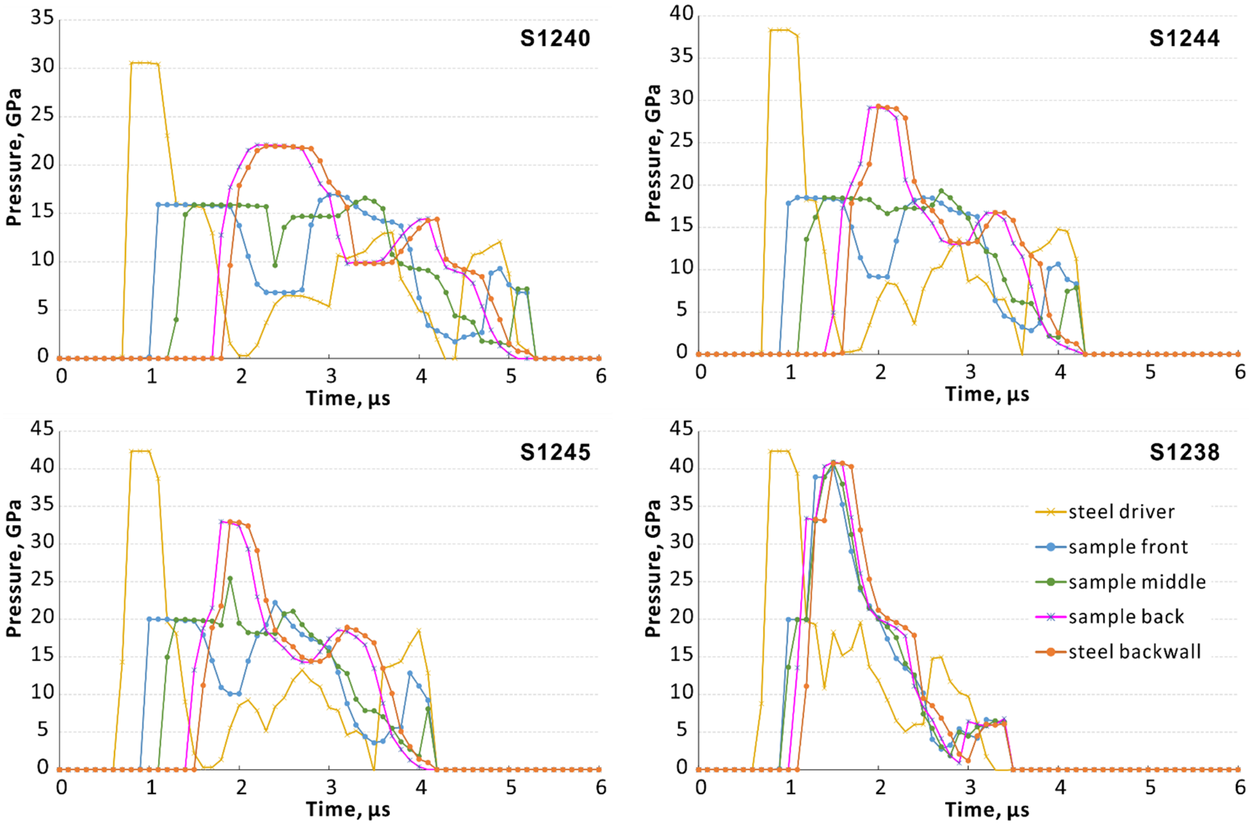

Fig. S5. P-t profiles computed with WONDY hydrocode for non-reverberating recovery shots (S1240, S1244, S1245) and one representative reverberation shot (S1238). Yellow and orange lines are profiles for the SS304 driver and back-wall, for reference. Blue, green and purple lines are for the front, middle, and back (in sequence along the impact direction) portions of the sample.

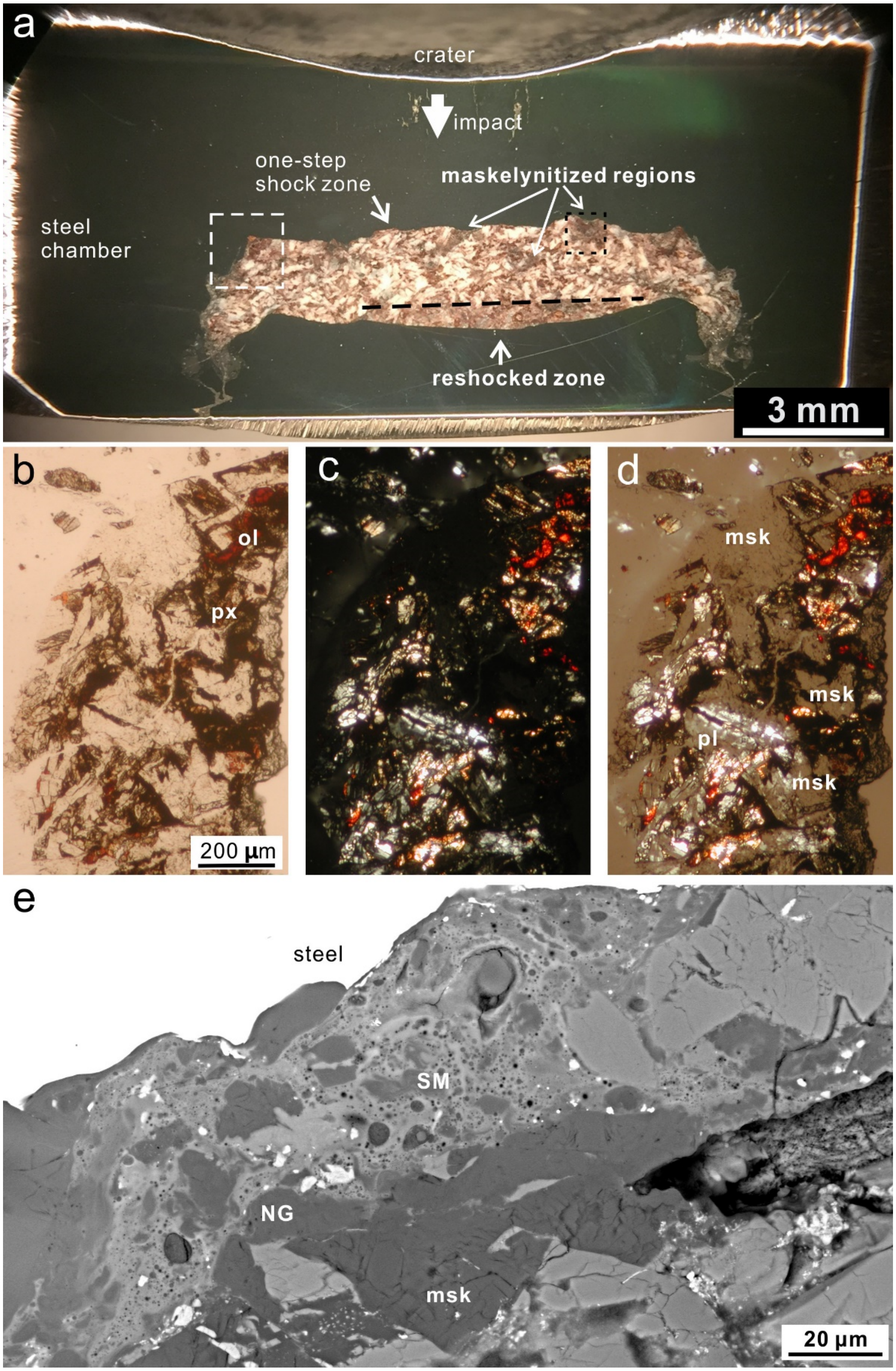

Fig. S6. Micrographs of recovery sample S1244 (single shock peak P of 17.4 GPa). Impact direction is top to bottom. (a) S1244 sample showing zones of transparent maskelynite and white-colored plagioclase. The reshocked zone contains only maskelynite. Maskelynite in thin

section shown by Figure 3c of the main text roughly corresponds to the area in the black dashed-box. (b-c) Images of the white dashed-box area in (a) under plane-polarized, cross-polarized and nonorthogonal (~87 °) cross-polarized light. Most plagioclase grains near the sample edge are fully converted to maskelynite (msk). Pyroxene (px) and altered olivine (ol; dark red) are less disturbed. The corner of the sample shown by the dashed-box may experience higher single-shock pressure and stronger shear than the central region due to the proximity of the high-impedance steel wall. Other areas near the front of the capsule may have also experienced slightly excess pressure due to convergent flow as the driver-sample interface deformed. (e) BSE image of a local shock melt (SM) pocket with features of flow and smearing, plus feldspathic normal glass (NG) with a slight schlieren effect. Maskelynite domains formed by solid-state amorphization show more factures than the smoother normal glass.

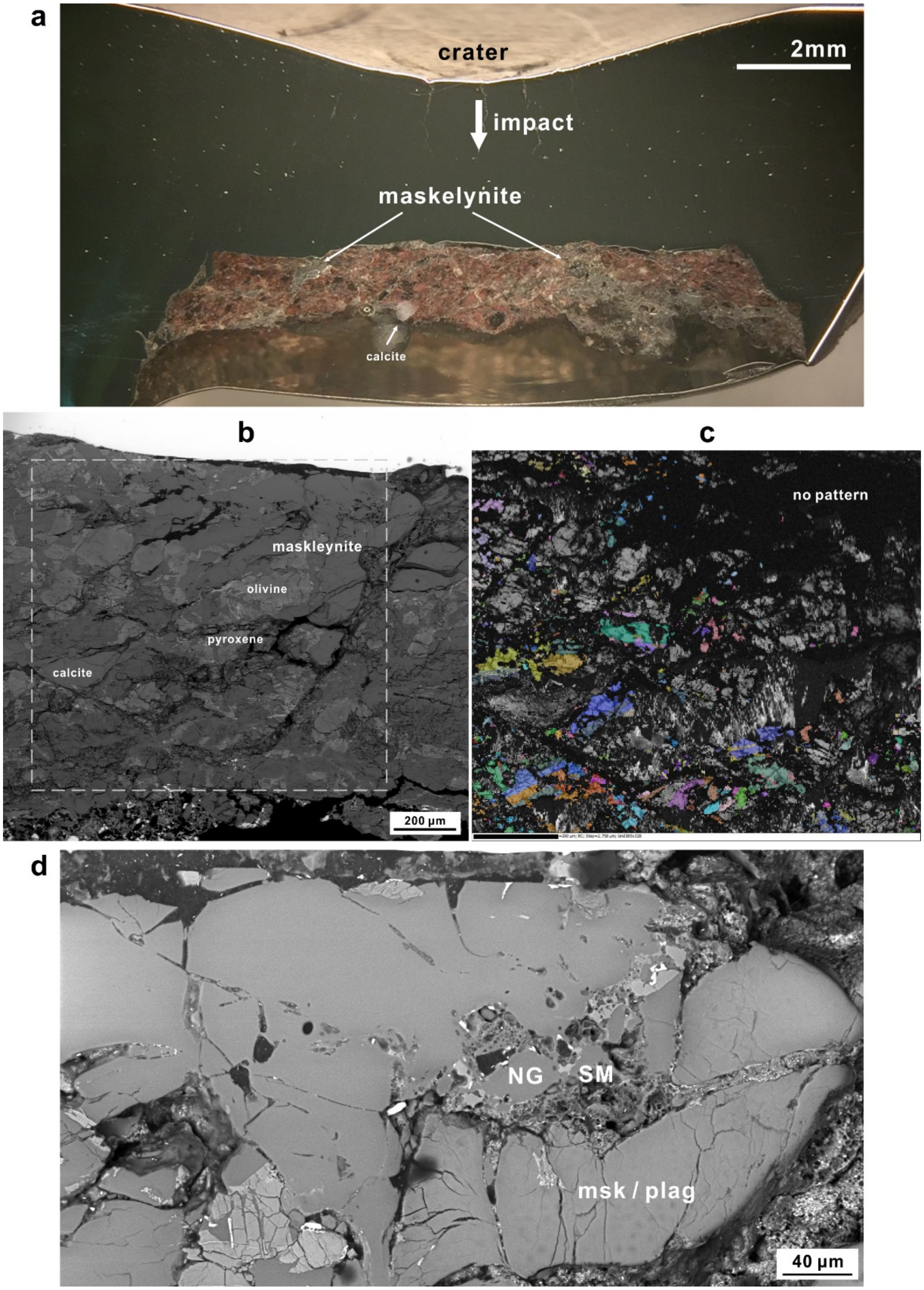

Fig. S7. Recovered sample of shot S1245 (single shock peak P of 19.3 GPa). Impact direction is top to bottom. (a) Thick section showing transparent feldspathic domains in maskelynitized zone. The back-wall cap is detached. (b) BSE image in variable-pressure mode, showing rock-forming minerals. The boxed area is mapped with EBSD. (c) The EBSD mapping result. Grey-scale brightness shows band contrast, a measure of the quality of the diffraction pattern and an

indicator of crystallinity. Dark areas indicate no pattern, meaning the surface is effectively amorphous. The colors demonstrate the variation in crystal orientation of plagioclase. EBSD contrast proves to be more sensitive to the presence of weak crystallinity, showing patterns in areas that appear fully isotropic to optical microscopy. (d) BSE image of local shock melt (SM) and feldspathic normal glass (NG). The adjacent unmelted maskelynite (msk)-plagioclase (plag) domains have higher density of fractures.

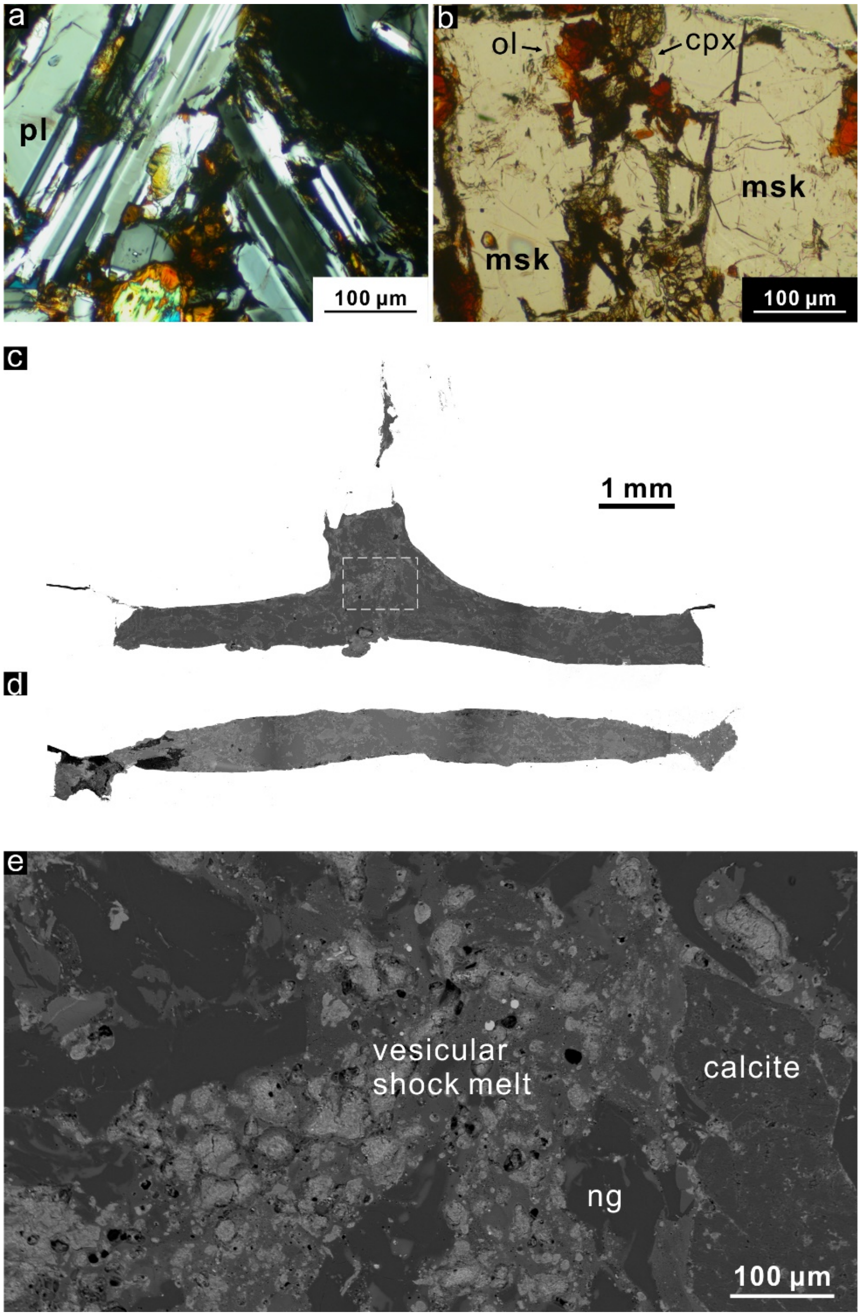

Fig. S8. Images of Saddleback basalt and reverberating experiments. (a) Cross-polarized light image of preshot Saddleback basalt showing laths of twinned labradorite. (b) Plane-polarized light image of full-reverberation shot S1232 (40.5 GPa) with uniformly complete maskelynite (msk) pseudomorphing labradorite, brown olivine (ol) and clinopyroxene (cpx). (c) BSE image of shot S1238 (full-reverberation pressure 42.4 GPa) showing central upwelling that nearly breached the crater floor. The melt pocked in dashed box is shown in e. (d) BSE image of shot S1236 (full-reverberation pressure 39.7 GPa). Impact direction is from top to bottom. (e) The BSE image of vesicular shock melt pockets in S1238, surrounded by calcite and feldspathic normal glass (ng).

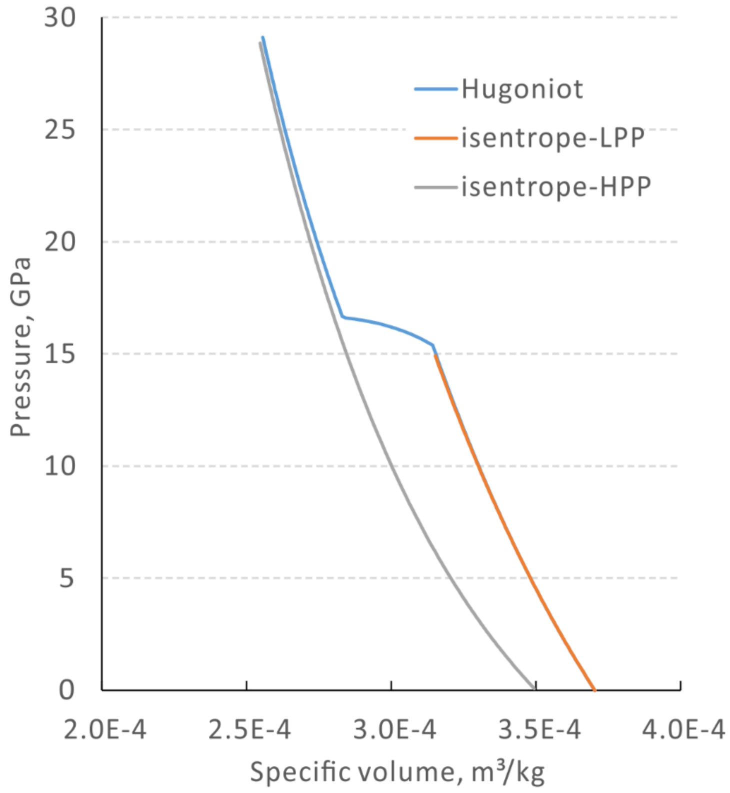

Fig. S9. The pressure-volume relationship of Saddleback basalt and the reference isentrope of initial (LPP) and high-pressure (HPP) state. The energy difference between LPP and HPP at zero-pressure is given by ΔE_{tr}. The energy difference between the Hugoniot (E_h) and isentrope (E_s) is given by E_v (see Table S4 and section S4).

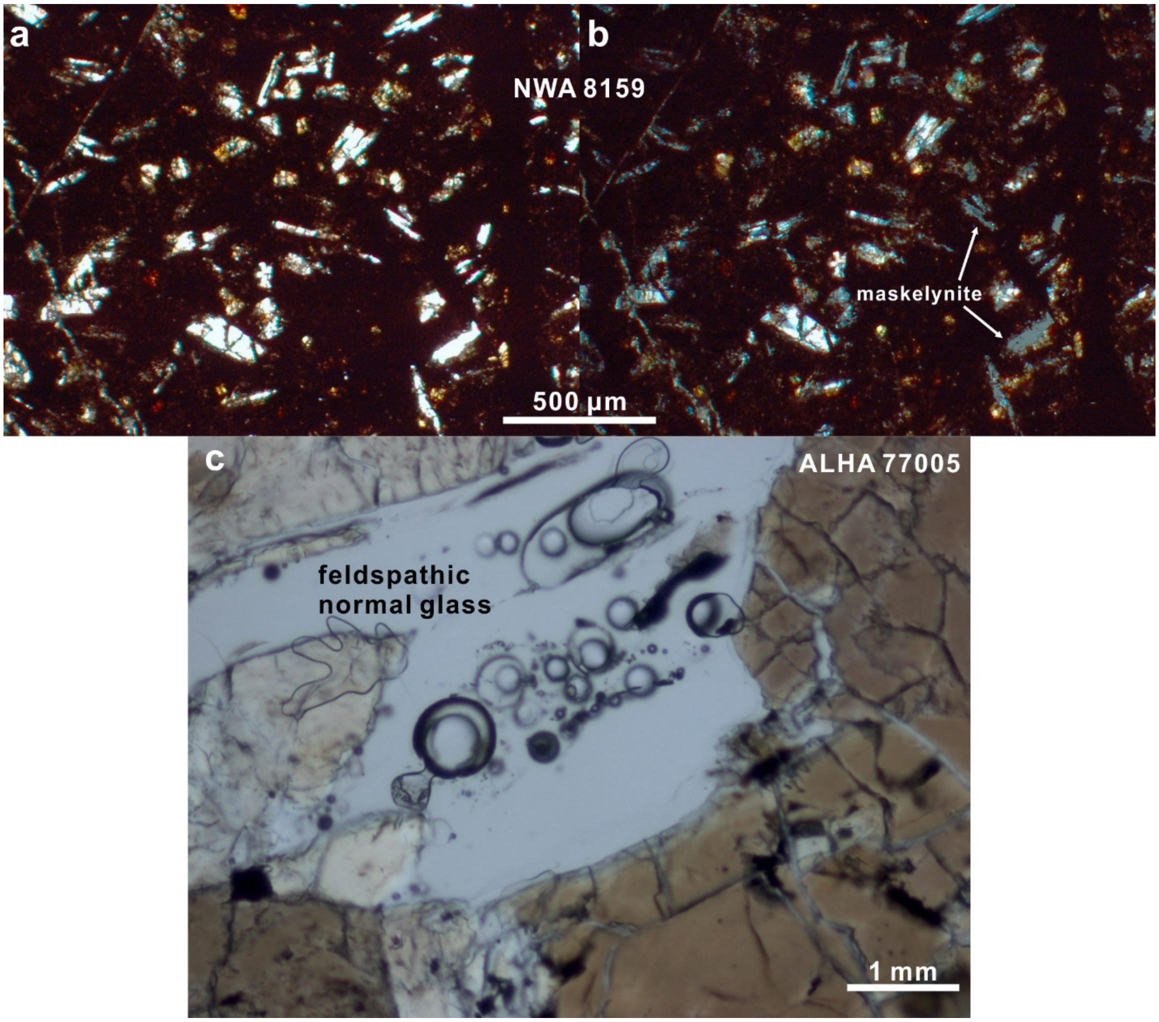

Fig. S10. Micrograph of shocked feldspars in martian meteorites. (a) low-magnification plane-polarized-light (PPL) image of NWA 8159 with multiple domains of feldspars (bright). (b) Nonorthogonal (87-88°) cross-polarized light cross-polarized-light image of the same area in (a), showing roughly half of the feldspar is isotropic (maskelynite) and the other half is birefringent. The polarizers are set to nonorthogonal to improve the contrast between maskelynite and matrix. (c) PPL of extensively melted shergottite ALHA77005. The feldspars are normal glass commonly with vesicles, suggesting quenching at low pressure after total remelting.

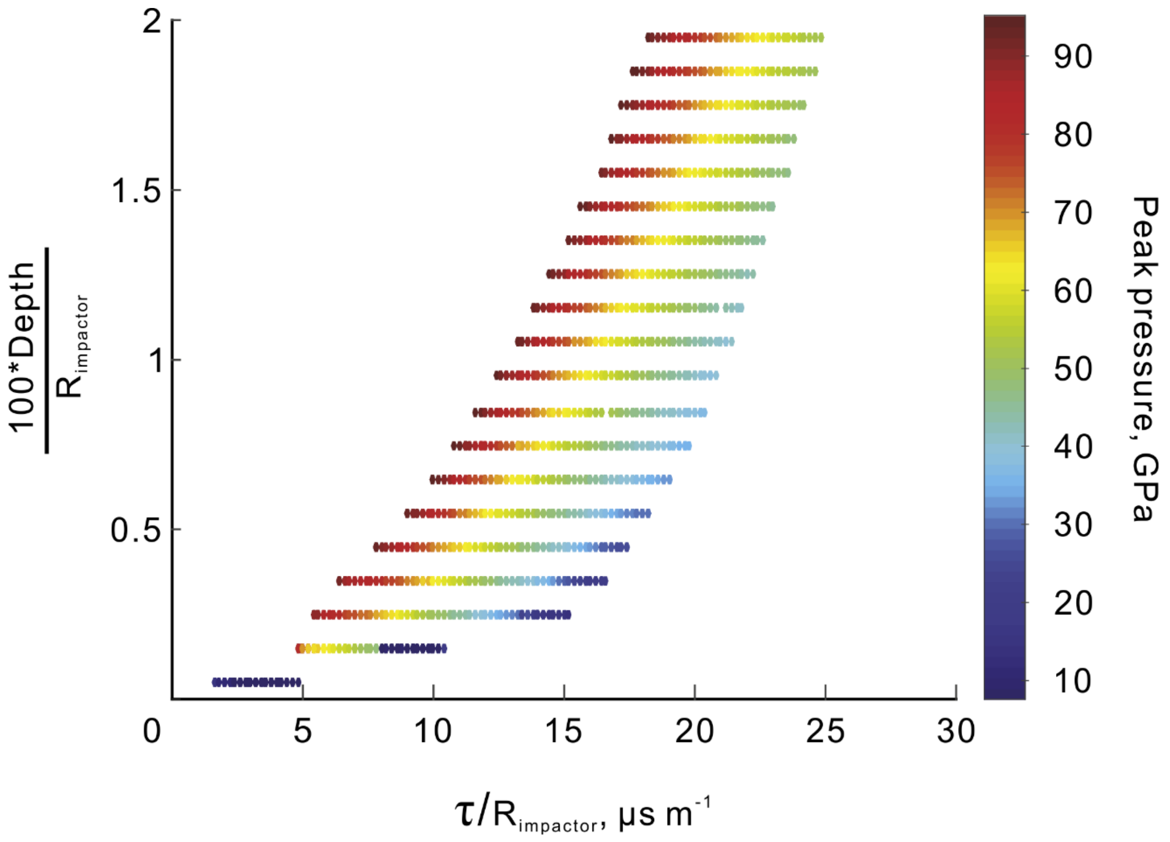

Fig. S11. Origin depth, dwell time and peak pressure of tracer particles ejected above escape velocity in modeled martian impacts (modified after Figure 8 in Bowling et al. (*8*). The y-axis is the origin depth of ejected particles, normalized to $1/100^{th}$ of $R_{impactor}$, the radius of the impactor. The x-axis is the dwell time of particles at > 1 GPa pressure, also normalized to $R_{impactor}$. For a lower peak pressure, the upper limit of dwell-time/$R_{impactor}$ is also lower, meaning a larger impactor is needed to sustain a given shock pulse.

	SiO_2	TiO_2	Al_2O_3	FeO*	MgO	CaO	Na_2O	K_2O	Cr_2O_3	MnO	CO_2	Total	vol %
labradorite	50.70	0.06	30.71	0.39	0.18	13.88	3.71	0.10	N.D.	N.D.	N.A.	99.73	58.6
andesine	59.40	0.16	24.80	0.97	0.06	7.12	7.23	0.38	0.03	N.D.	N.A.	100.15	5.0
magnesian olivine	39.24	0.01	0.04	19.81	39.90	0.30	N.D.	0.01	0.07	0.29	N.A.	99.68	10.4
hortonolite olivine	35.17	0.10	0.03	35.39	27.36	0.27	N.D.	N.D.	0.01	0.63	N.A.	98.99	3.0
ferrous augite	48.66	1.54	5.30	24.93	3.81	13.12	1.12	0.05	N.D.	0.79	N.A.	99.33	6.0
diopside	48.58	2.59	4.28	11.10	11.33	21.13	0.37	0.02	0.27	0.20	N.A.	99.88	10.0
calcite	N.D.	N.D.	N.D.	N.D.	0.25	55.78	N.D.	N.D.	N.D.	N.D.	44.04	100.07	4.7
hematite	0.12	1.06	1.84	91.17	0.70	0.11	0.01	0.01	4.40	0.74	N.A.	100.15	1.5
altered ilmenite	0.31	48.80	0.05	40.71	2.67	0.20	0.01	0.03	0.02	1.36	N.A.	94.17	0.7
	Uncertainties (1σ)												
labradorite	0.17	0.03	0.13	0.03	0.02	0.07	0.07	0.01	-	-	-		
andesine	0.19	0.03	0.12	0.04	0.01	0.05	0.10	0.01	0.03	-	-		
magnesian olivine	0.16	0.03	0.02	0.14	0.17	0.01	-	0.01	0.03	0.03	-		
ferrous olivine	0.15	0.03	0.02	0.19	0.15	0.01	-	0.01	0.03	0.03	-		
ferrous augite	0.17	0.05	0.06	0.16	0.06	0.07	0.04	0.01	0.03	0.04	-		
diopside	0.17	0.06	0.05	0.11	0.09	0.08	0.03	0.01	0.03	0.03	-		
calcite	-	-	-	-	0.02	0.13	-	-	-	-	0.10		
hematite	0.03	0.04	0.04	0.30	0.03	0.01	0.02	0.01	0.06	0.04	-		
altered ilmenite	0.03	0.22	0.02	0.20	0.05	0.01	0.02	0.01	0.03	0.04	-		

Table S1. Representative EPMA analyses and modal percentages of rock forming minerals in Saddleback basalt.

Concentrations are in weight percent (wt %), processed with CITZAF procedure. *Total Fe is reported as FeO except that Fe_2O_3 is reported for hematite. CO_2 content in calcite is calculated assuming all measured cations form carbonate and is not applicable (N.A.) for other minerals. Elements below detection limits are shown as not detected (N.D.). The detection limits for listed elements are between 0.01-0.03 wt %. Titanium oxide (ilmenite) commonly shows 1:1 Fe/Ti atomic ratio and total below 100 wt%, inferred to result from aqueous alteration.

	SiO_2	TiO_2	Al_2O_3	FeO*	MgO	CaO	Na_2O	K_2O	Cr_2O_3	MnO	P_2O_5	LOI	Total
[1]Model	45.3	1.3	18.1	9.6	7.8	13.4	2.3	0.1	0.1	0.1		1.8	100.0
[2]XRF	47.7	0.9	16.5	10.8	6.5	10.3	2.5	0.4	0.0	0.2	0.2	2.8	98.8
	47.8	1.1	16.7	10.7	7.2	10.6	3.4	0.5	0.1	0.2	0.2	1.2	99.6
	49.7	1.0	16.8	10.7	4.7	9.7	3.2	0.5	0.0	0.2	0.2	2.2	98.7
	47.2	1.2	16.8	10.6	5.7	11.5	3.6	0.4	0.1	0.2	0.2	2.3	99.7
	47.6	1.1	16.1	9.8	6.5	10.5	3.4	0.4	0.1	0.2	0.3	1.7	97.6
	47.7	1.0	17.4	11.3	6.1	10.2	3.3	0.6	0.1	0.2	0.2	2.1	100.0
	47.5	1.2	16.4	10.4	4.8	10.3	4.1	0.6	0.1	0.2	0.1	5.3	100.0
	48.2	1.2	17.1	10.8	5.4	10.3	3.0	0.3	0.1	0.2	0.1	3.1	99.6
	47.1	0.9	15.5	9.7	6.1	7.9	2.1	0.5	0.0	0.2	0.1	9.8	99.8
	47.1	0.9	16.1	10.3	5.9	8.6	2.3	0.4	0.1	0.1	0.1	7.8	99.7
[3]NWA 8159	47	0.65	10.74	20.86	4.35	11.98	2.46	0.10	0.14	0.46	0.26		100

Table S2. Bulk composition of Saddleback basalt and shergottite NWA 8159.

Concentrations are in weight percentage. *XRF results assume pure ferric ion. The FeO data from EPMA is recalculated to Fe_2O_3 for easy comparison. [1]Mixing model based on mineral compositions and contents from Table S1; LOI (loss on ignition) in the model is estimated using the CO_2 content. [2]Peters et al. (*29*); [3]Herd et al. (*60*).

recovery shot no.	flyer material	chamber material	impact velocity, m s^{-1}	flyer thickness, mm	sample thickness, mm	first shock pressure, GPa	reflected shock pressure, GPa	sample-driver peak pressure, GPa	labradorite one shock	labradorite reshock	labradorite reverberation
1240	Ta	SS304	1156	2.08	5.01	15.8	21.8	N.A.*	pl	msk	
1239	Ta	SS304	1221	2.05	1.37	16.2	23.7	32.7			msk
1244	Ta	SS304	1399	1.57	3.78	17.4	29.2	N.A.	pl-msk	msk	
1236	Ta	SS304	1438	2.00	1.00	18.0	30.2	39.7			msk
1232	Ta	SS304	1463	2.08	0.98	18.4	30.9	40.5			msk
1238	Ta	SS304	1519	1.58	1.10	19.3	32.4	42.4			ng
1245	Ta	SS304	1521	1.54	3.64	19.3	32.4	N.A.	msk-pl	msk	

EoS shot no.	flyer material	driver plate material	impact velocity, m s^{-1}	sample bulk density, g cm^{-3}	first shock velocity, m s^{-1}	first particle velocity, m s^{-1}	first free surface velocity, m s^{-1}	first shock pressure, GPa	final shock velocity, m s^{-1}	final free surface velocity, m s^{-1}	final particle velocity, m s^{-1}	final shock pressure, GPa
1123	Ta	Ta	1394	2.7	6137 ± 72	900 [a]	N.M.	15.4	N.M.		N.M.	-
1126	Ta	Ta	1479	2.7	5766 ± 87	1028	2056	16.0	5115 [b]	3072	1203	16.6
1124	Ta	Ta	1598	2.7	5233 $_{-57}^{+180}$ [c]	1297		18.3	-		-	-

Table S3. Summary of Shock EoS and recovery experiments

*Peak driver-sample pressure is not applicable (N.A.) for recovery experiments with thick samples that do not reach sample-driver pressure equilibrium. Abbreviation: plagioclase (pl), maskelynite (msk), normal glass (ng). a The particle velocity of shot 1124 is loosely constrained by assuming linearity in Us-up at lower pressure than shot 1126. b The uncertainty of the final shock velocity is the same as first shock velocity, caused by sample heterogeneity. c The asymmetrical uncertainty results from the heterogeneity of the sample plus the variation of shock arrival from tilt of shock front. The corresponding uncertainties in pressure are presented in Fig. S2. Shot 1123 uses a flat mirror setup whose first-wave velocity and particle velocities are not measured (N.M.).

	San Gabriel Anorthosite[a]	Vacaville basalt[b]	Saddleback basalt low-density	Saddleback basalt mixed	Saddleback basalt high-density
ρ_o, kg m^{-3}	2662	2820	2700	2700	2857
V_o, m^3 kg^{-1}	3.76E–04	3.55E–04	3.70E–04	3.70E–04	3.50E–04[d]
K_{os}, Pa	3.79E+10	5.61E+10	7.37E+10		4.78E+10
K_s'	4.73	2.28	2.93		4.03
γ_o	2.2	1.39	1.39	1.39	1.44
q	1.66	1	1	1	1.7
ν	0.25	0.25	0.25	0.25	0.25
C_o, m s^{-1}	3773	4462	5224[c]	9594	3604
s	1.43	0.82	0.98	-3.72	1.26
C_p, J kg^{-1} K^{-1}	$a+bT^{-1}+cT^{-2}+dT^{-3}$	$a+bT^{-1}+cT^{-2}+dT^{-0.5}$			
a	439.37	2.337			
b	–3.73E–01	–2.773E–4			
c	0	22020			
d	-3.17E–06	–29.76			
ΔE_{tr}, J/kg					2.41E+05[e]
impedance, kg s^{-1} m^{-2}	1.00E+07	1.26E+07	1.41E+07	2.59E+07	1.03E+07
references	1, 2	1, 3, 4	3, 4, 7	3, 4	3, 5, 6

Table S4. Hugoniot and thermodynamic parameters for shock-temperature calculations

[a] for comparison to labradorite shock-recovery; [b] The petrography of Vacaville basalt is similar to the Lonar basalt used in shock-recovery experiments by Kieffer et al. (*11*) (Fig. 1 in the main text); [c] sound speed and bulk modulus of Saddleback basalt at ambient pressure (low-density regime) are from Peters et al. (*29*); The properties of Saddleback basalt for mixed and high-density regime in shocked state are obtained by shock experiments from this study [d] assuming same volume difference between low- and high-pressure phases as at transition pressure (Fig. S9); [e] assuming 60% plagioclase in basalt.

References: Hugoniot, Ahrens and Gregson (*37*); heat capacity, Berman (*61*), Bouhifd et al. (*62*); Grüneisen parameter, Ahrens and O'Keefe (*63*), Boslough et al. (*58*); Transition enthalpy, Navrotsky (*57*); Sound speed, Peters et al. (*29)*

REFERENCES AND NOTES

1. A. E. Rubin, Maskelynite in asteroidal, lunar and planetary basaltic meteorites: An indicator of shock pressure during impact ejection from their parent bodies. *Icarus* **257**, 221–229 (2015).

2. G. Tschermak, Die Meteoriten von Schergotty und Gopalpur. *Sitz-Ber. K. Akad. Wiss., math.-naturw. Kl.* **65**, 122–146 (1872).

3. L. Ferrière, F. Brandstätter, What is maskelynite? Back to the original description and thin sections in which it was first described. *78th Annual Meeting of the Meteoritical Society.* **50**, 5184 (2015).

4. D. J. Milton, P. S. de Carli, Maskelynite: Formation by explosive shock. *Science* **140**, 670–671 (1963).

5. D. E. Moser, K. R. Chamberlain, K. T. Tait, A. K. Schmitt, J. R. Darling, I. R. Barker, B. C. Hyde, Solving the martian meteorite age conundrum using micro-baddeleyite and launch-generated zircon. *Nature* **499**, 454–457 (2013).

6. J. N. Head, H. J. Melosh, B. A. Ivanov, Martian meteorite launch: High-speed ejecta from small craters. *Science* **298**, 1752–1756 (2002).

7. K. Kurosawa, T. Okamoto, H. Genda, Hydrocode modeling of the spallation process during hypervelocity impacts: Implications for the ejection of martian meteorites. *Icarus* **301**, 219–234 (2018).

8. T. J. Bowling, B. C. Johnson, S. E. Wiggins, E. L. Walton, H. J. Melosh, T. G. Sharp, Dwell time at high pressure of meteorites during impact ejection from Mars. *Icarus* **343**, 113689 (2020).

9. D. Stöffler, R. Ostertag, C. Jammes, G. Pfannschmidt, P. R. S. Gupta, S. B. Simon, J. J. Papike, R. H. Beauchamp, Shock metamorphism and petrography of the Shergotty achondrite. *Geochim. Cosmochim. Acta* **50**, 889–903 (1986).

10. J. Fritz, A. Greshake, D. Stöffler, Micro-Raman spectroscopy of plagioclase and maskelynite in martian meteorites: Evidence of progressive shock metamorphism. *Antarct. Meteor. Res.* **18**, 96 (2005).

11. S. W. Kieffer, R. B. Schaal, R. Gibbons, R. Horz, D. J. Milton, A. Dube, Shocked basalt from Lonar impact crater, India, and experimental analogues. *Lunar and Planetary Science Conference Proceedings* **1**, 1391–1412 (1976).

12. R. V. Gibbons, T. J. Ahrens, Effects of shock pressures on calcic plagioclase. *Phys. Chem. Minerals* **1**, 95–107 (1977).

13. R. Ostertag, Shock experiments on feldspar crystals. *J. Geophys. Res.* **88**, B364–B376 (1983).

14. J. R. Johnson, F. Hörz, Visible/near-infrared spectra of experimentally shocked plagioclase feldspars. *J. Geophys. Res.* **108**, 5120 (2003).

15. J. Fritz, V. Assis Fernandes, A. Greshake, A. Holzwarth, U. Böttger, On the formation of diaplectic glass: Shock and thermal experiments with plagioclase of different chemical compositions. *Meteorit. Planet. Sci.* **54**, 1533–1547 (2019).

16. N. Tomioka, M. Miyahara, High-pressure minerals in shocked meteorites. *Meteorit. Planet. Sci.* **52**, 2017–2039 (2017).

17. T. G. Sharp, E. L. Walton, J. Hu, C. Agee, Shock conditions recorded in NWA 8159 martian augite basalt with implications for the impact cratering history on Mars. *Geochim. Cosmochim. Acta* **246**, 197–212 (2019).

18. E. L. Walton, C. D. K. Herd, Dynamic crystallization of shock melts in Allan Hills 77005: Implications for melt pocket formation in martian meteorites. *Geochim. Cosmochim. Acta* **71**, 5267–5285 (2007).

19. O. Ohtaka, H. Fukui, T. Kunisada, T. Fujisawa, K. Funakoshi, W. Utsumi, T. Irifune, K. Kuroda, T. Kikegawa, Phase relations and equations of state of ZrO_2 under high temperature and high pressure. *Phys. Rev. B* **63**, 174108 (2001).

20. L. F. White, J. R. Darling, D. E. Moser, C. Cayron, I. Barker, J. Dunlop, K. T. Tait, Baddeleyite as a widespread and sensitive indicator of meteorite bombardment in planetary crusts. *Geology* **46**, 719–722 (2018).

21. T. Kubo, M. Kimura, T. Kato, M. Nishi, A. Tominaga, T. Kikegawa, K.-i. Funakoshi, Plagioclase breakdown as an indicator for shock conditions of meteorites. *Nat. Geosci.* **3**, 41–45 (2010).

22. A. R. Huffman, W. U. Reimold, Experimental constraints on shock-induced microstructures in naturally deformed silicates. *Tectonophysics* **256**, 165–217 (1996).

23. M. Sims, S. J. Jaret, E.-R. Carl, B. Rhymer, N. Schrodt, V. Mohrholz, J. Smith, Z. Konopkova, H.-P. Liermann, T. D. Glotch, L. Ehm, Pressure-induced amorphization in plagioclase feldspars: A time-resolved powder diffraction study during rapid compression. *Earth Planet. Sci. Lett.* **507**, 166–174 (2019).

24. A. E. Gleason, S. Park, D. R. Rittman, A. Ravasio, F. Langenhorst, R. M. Bolis, E. Granados, S. Hok, T. Kroll, M. Sikorski, T.-C. Weng, H. J. Lee, B. Nagler, T. Sisson, Z. Xing, D. Zhu, G. Giuli, W. L. Mao, S. H. Glenzer, D. Sokaras, R. Alonso-Mori, Ultrafast structural response of shock-compressed plagioclase. *Meteorit. Planet. Sci.* **57**, 635–643 (2022).

25. J. Hu, T. G. Sharp, Formation, preservation and extinction of high-pressure minerals in meteorites: Temperature effects in shock metamorphism and shock classification. *Prog. Earth Planet. Sci.* **9**, 6 (2022).

26. J.-G. Moreau, T. Kohout, K. Wünnemann, Melting efficiency of troilite-iron assemblages in shock-darkening: Insight from numerical modeling. *Phys. Earth Planet. In.* **282**, 25–38 (2018).

27. T. G. Sharp, P. S. DeCarli, Shock effects in meteorites in *Meteorites and the early solar system II*, D. S. Lauretta, H. Y. McSween Jr., Eds. (The University of Arizona Press, Tucson, 2006), 653–677.

28. Y. Liu, Y. Chen, Y. Guan, C. Ma, G. R. Rossman, J. M. Eiler, Y. Zhang, Impact-melt hygrometer for Mars: The case of shergottite Elephant Moraine (EETA) 79001. *Earth Planet. Sci. Lett.* **490**, 206–215 (2018).

29. G. H. Peters, W. Abbey, G. H. Bearman, G. S. Mungas, J. A. Smith, R. C. Anderson, S. Douglas, L. W. Beegle, Mojave Mars Simulant—Characterization of a new geologic Mars analog. *Icarus* **197**, 470–479 (2008).

30. S. Nakazawa, S. Watanabe, M. Kato, Y. Iijima, T. Kobayashi, T. Sekine, Hugoniot equation of state of basalt. *Planet. Space Sci.* **45**, 1489–1492 (1997).

31. T. Sekine, T. Kobayashi, M. Nishio, E. Takahashi, Shock equation of state of basalt. *Earth Planets Space* **60**, 999–1003 (2008).

32. I. Daniel, P. Gillet, P. F. McMillan, G. Wolf, M. A. Verhelst, High-pressure behavior of anorthite: Compression and amorphization. *J. Geophys. Res.* **102**, 10313–10325 (1997).

33. P. Beck, P. Gillet, A. El Goresy, S. Mostefaoui, Timescales of shock processes in chondritic and martian meteorites. *Nature* **435**, 1071–1074 (2005).

34. E. L. Walton, T. G. Sharp, J. Hu, J. Filiberto, Heterogeneous mineral assemblages in martian meteorite Tissint as a result of a recent small impact event on Mars. *Geochim. Cosmochim. Acta* **140**, 334–348 (2014).

35. J. Fritz, A. Greshake, High-pressure phases in an ultramafic rock from Mars. *Earth Planet. Sci. Lett.* **288**, 619–623 (2009).

36. A. Takenouchi, T. Mikouchi, T. Kobayashi, T. Sekine, A. Yamaguchi, H. Ono, Fine-structures of planar deformation features in shocked olivine: A comparison between martian

meteorites and experimentally shocked basalts as an indicator for shock pressure. *Meteorit. Planet. Sci.* **54**, 1990–2005 (2019).

37. T. J. Ahrens, V. Gregson Jr., Shock compression of crustal rocks: Data for quartz, calcite, and plagioclase rocks. *J. Geophys. Res.* **69**, 4839–4874 (1964).

38. N. K. Bourne, J. C. F. Millett, J. E. Field, On the strength of shocked glasses. *Proc. R. Soc. Lond. A* **455**, 1275–1282 (1999).

39. F. Langenhorst, P. Joreau, J. C. Doukhan, Thermal and shock metamorphism of the Tenham chondrite: A TEM examination. *Geochim. Cosmochim. Acta* **59**, 1835–1845 (1995).

40. A. S. P. Rae, M. H. Poelchau, T. Kenkmann, Stress and strain during shock metamorphism. *Icarus* **370**, 114687 (2021).

41. C. T. Adcock, O. Tschauner, E. M. Hausrath, A. Udry, S. N. Luo, Y. Cai, M. Ren, A. Lanzirotti, M. Newville, M. Kunz, C. Lin, Shock-transformation of whitlockite to merrillite and the implications for meteoritic phosphate. *Nat. Commun.* **8**, 14667 (2017).

42. A. Greshake, J. Fritz, U. Böttger, D. Goran, Shear-induced ringwoodite formation in the martian shergottite Dar al Gani 670. *Earth Planet. Sci. Lett.* **375**, 383–394 (2013).

43. S. Shkolyar, S. J. Jaret, B. A. Cohen, J. R. Johnson, O. Beyssac, J. M. Madariaga, R. C. Wiens, A. Ollila, S. Holm-Alwmark, Y. Liu, Identifying shocked feldspar on Mars using perseverance spectroscopic instruments: Implications for geochronology studies on returned samples. *Earth Moon Planets* **126**, 4 (2022).

44. E. L. Walton, S. P. Kelley, C. D. K. Herd, Isotopic and petrographic evidence for young martian basalts. *Geochim. Cosmochim. Acta* **72**, 5819–5837 (2008).

45. H. Leroux, W. Reimold, C. Koeberl, U. Hornemann, J.-C. Doukhan, Experimental shock deformation in zircon: A transmission electron microscopic study. *Earth Planet. Sci. Lett.* **169**, 291–301 (1999).

46. L. G. Staddon, J. R. Darling, W. H. Schwarz, N. R. Stephen, S. Schuindt, J. Dunlop, K. T. Tait, Dating martian mafic crust; microstructurally constrained baddeleyite geochronology of enriched shergottites Northwest Africa (NWA) 7257, NWA 8679 and Zagami. *Geochim. Cosmochim. Acta* **315**, 73–88 (2021).

47. T. Niihara, H. Kaiden, K. Misawa, T. Sekine, T. Mikouchi, U–Pb isotopic systematics of shock-loaded and annealed baddeleyite: Implications for crystallization ages of martian meteorite shergottites. *Earth Planet. Sci. Lett.* **341–344**, 195–210 (2012).

48. J. R. Elliott, H. J. Melosh, B. C. Johnson, The role of target strength on the ejection of martian meteorites. *Icarus* **375**, 114869 (2022).

49. P. H. Warren, Lunar and martian meteorite delivery services. *Icarus* **111**, 338–363 (1994).

50. A. Lagain, G. K. Benedix, K. Servis, D. Baratoux, L. S. Doucet, A. Rajšic, H. A. R. Devillepoix, P. A. Bland, M. C. Towner, E. K. Sansom, K. Miljković, The Tharsis mantle source of depleted shergottites revealed by 90 million impact craters. *Nat. Commun.* **12**, 6352 (2021).

51. R. Wieler, L. Huber, H. Busemann, S. Seiler, I. Leya, C. Maden, J. Masarik, M. M. M. Meier, K. Nagao, R. Trappitsch, A. J. Irving, Noble gases in 18 martian meteorites and angrite Northwest Africa 7812—Exposure ages, trapped gases, and a re-evaluation of the evidence for solar cosmic ray-produced neon in shergottites and other achondrites. *Meteorit. Planet. Sci.* **51**, 407–428 (2016).

52. K. Hirose, Y. Fei, Y. Ma, H.-K. Mao, The fate of subducted basaltic crust in the Earth's lower mantle. *Nature* **397**, 53–56 (1999).

53. A. Takenouchi, A. Yamaguchi, T. Mikouchi, A Newly Found Unique Shergottite Asuka 12325: Comparison in Petrology and Shock Metamorphism with Other Poikilitic Shergottites, *Lunar and Planetary Science Conference LI*, 1326 (2020).

54. J. D. Piercy, J. C. Bridges, L. J. Hicks, J. L. MacArthur, R. C. Greenwood, I. A. Franchi, Terrestrial alteration mineral assemblages in the NWA 10416 olivine phyric shergottite. *Geochim. Cosmochim. Acta* **280**, 26–45 (2020).

55. T. J. Ahrens, C. F. Petersen, J. T. Rosenberg, Shock compression of feldspars. *J. Geophys. Res.* **74**, 2727–2746 (1969).

56. M. E. Kipp, R. J. Lawrence, WONDY V: A one-dimensional finite-difference wave-propagation code. *NASA STI/Recon Technical Report N.* **83** (1982)

57. A. Navrotsky, R. Hon, D. F. Weill, D. J. Henry, Thermochemistry of glasses and liquids in the systems $CaMgSi_2O_6$-$CaAl_2Si_2O_8$-$NaAlSi_3O_8$, SiO_2-$CaAl_2Si_2O_8$-$NaAlSi_3O_8$ and SiO_2-Al_2O_3-CaO-Na_2O. *Geochim. Cosmochim. Acta* **44**, 1409–1423 (1980).

58. M. B. Boslough, S. M. Rigden, T. J. Ahrens, Hugoniot equation of state of anorthite glass and lunar anorthosite. *Geophys. J. Roy. Astron. Soc.* **84**, 455–473 (1986).

59. R. G. McQueen, S. P. Marsh, J. N. Fritz, Hugoniot equation of state of twelve rocks. *J. Geophys. Res.* **72**, 4999–5036 (1967).

60. C. D. K. Herd, E. L. Walton, C. B. Agee, N. Muttik, K. Ziegler, C. K. Shearer, A. S. Bell, A. R. Santos, P. V. Burger, J. I. Simon, M. J. Tappa, F. M. McCubbin, J. Gattacceca, F. Lagroix, M. E. Sanborn, Q.-Z. Yin, W. S. Cassata, L. E. Borg, R. E. Lindvall, T. S. Kruijer, G. A. Brennecka, T. Kleine, K. Nishiizumi, M. W. Caffee, The Northwest Africa 8159 martian meteorite: Expanding the martian sample suite to the early Amazonian. *Geochim. Cosmochim. Acta* **218**, 1–26 (2017).

61. R. G. Berman, Internally-consistent thermodynamic data for minerals in the system Na_2O-K_2O-CaO-MgO-FeO-Fe_2O_3-Al_2O_3-SiO_2-TiO_2-H_2O-CO_2. *J. Petrol.* **29**, 445–522 (1988).

62. M. A. Bouhifd, P. Besson, P. Courtial, C. Gérardin, A. Navrotsky, P. Richet, Thermochemistry and melting properties of basalt. *Contrib. Mineral. Petrol.* **153**, 689–698 (2007).

63. T. J. Ahrens, J. D. Okeefe, Equations of state and impact-induced shock-wave attenuation on the moon, in *Impact and Explosion Cratering: Planetary and Terrestrial Implications* (Pergamon Press, New York, 1977), 639–656.
